# Three new triterpenoids from *Rubia schumanniana*

**DOI:** 10.1007/s13659-012-0038-8

**Published:** 2012-07-12

**Authors:** Bin Kuang, Jing Han, Guang-Zhi Zeng, Xiao-Qiang Chen, Wen-Jun He, Ning-Hua Tan

**Affiliations:** 1 State Key Laboratory of Phytochemistry and Plant Resources in West China, Kunming Institute of Botany, Chinese Academy of Sciences, Kunming, 650201 China; 2 Graduate University of Chinese Academy of Sciences, Beijing, 100049 China

**Keywords:** *Rubia schumanniana*, triterpenoid, cytotoxicity

## Abstract

Three new triterpenoids, 3*β*-hydroxy-urs-30-*p-Z*-hydroxycinnamoyl-12-en-28-oic-acid (**1**), 3*β*-hydroxy-olean-30-*p-E*hydroxycinnamoyl-12-en-28-oic-acid (**2**) and 3*β*,6*α*-dihydroxy-urs-14-en-12-one (**3**), together with seven known triterpenoids, were isolated from the roots of *Rubia schumanniana*. Their structures were established by means of spectroscopic analysis. All compounds were evaluated for cytotoxic activity, and compounds **2–6** showed cytotoxicity with the IC50 values of 10.75∼18.87 µg/mL.

**Electronic Supplementary Material:**

Supplementary material is available for this article at 10.1007/s13659-012-0038-8 and is accessible for authorized users.

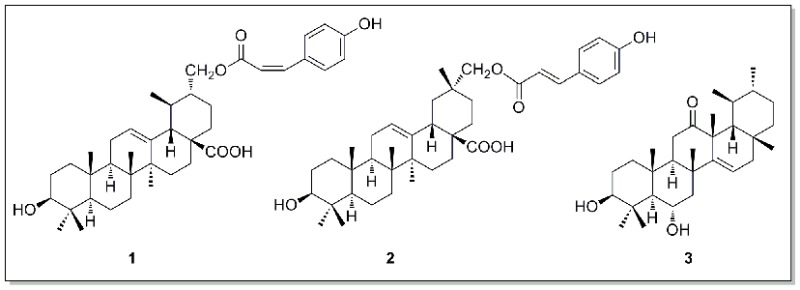

## Introduction

*Rubia schumanniana*, an endemic species, is mainly distributed in southwest China. As one of the substitutes of traditional Chinese medicine *R. cordifolia*, its roots have been used for the treatment of tuberculosis, rheumatism, contusion, febrility and menoxenia. Previous studies on this plant have resulted in the isolation of seven quinions and *β*-sitosterol.[1,2] As part of our continuing research on chemical constituents of medicinal plants from the genus *Rubia*, a systematic phytochemical investigation of the roots of *R. schumanniana* was carried out, which led to the isolation of three new triterpenoids (**1**–**3**), along with seven known triterpenoids, zamanic acid (**4**),[3] maslinic acid (**5**),[4] ursolic acid (**6**),[5] rubifolic acid (**7**),[6] oleanolic acid (**8**),[7] karachic acid (**9**),[8] and rubiarbonol K (**10**).[9] All compounds were evaluated for cytotoxicity against three human cancer cell lines (Hela, BGC-823, A549). Herein, we report the isolation, structural determination, and cytotoxic activities of these compounds.

## Results and Discussion

Compound **1** was obtained as a white powder with a positive specific rotation ($[\alpha]_D^{16}$ + 6.5). Its molecular formula, $C_{39}H_{54}O_6$, was deduced by HRESIMS (*m/z* 617.3856 $[M - H]^-$), indicating 13 degrees of unsaturation. The IR spectrum showed absorption bands for hydroxyl (3426 $cm^{-1}$), carbonyl (1689 $cm^{-1}$) and olefinic (1632 $cm^{-1}$) groups. The $^{13}C$ NMR spectrum of **1** (Table 1) exhibited 39 carbons, including one trisubstituted double bond ($\delta_C$ 126.7, 139.3), one carboxyl ($\delta_C$ 180.3) and one *p*-hydroxycinnamoyl group, six methyls, ten methylenes (one oxygenated), six methines (one oxygenated), and five quaternary carbons. Comparison of the NMR data of **1** with those of zamanic acid (**4**) revealed that both compounds are ursane-type triterpenoids. The only difference between them was that the coupling constant of the disubstituted double bond in *p*-hydroxycinnamoyl group is 13.0 Hz in **1** while 16.0 Hz in **4**. The HMBC correlations of H-30 with the ester carbonyl carbon, C-19, C-20, and C-21 enabled the *p*-*Z*-hydroxycinnamoyl group to be placed at C-30 (Figure 1). The relative configuration of **1** was deduced from the analysis of its ROESY spectrum (Figure 2). The observed NOE correlations of H-3/H-5 and Me-23, H-5/H-9, and H-9/Me-27 indicated that H-3, H-5, H-9, Me-23 and Me-27 are cofacial and assigned as *α*-oriented. In turn the cross-peaks of Me-25/Me-24 and Me-26, and H-20/H-18 and Me-29 indicated the *β*-oriented of H-18, H-20, Me-24, Me-25, Me-26 and Me-29. From the above evidences, the structure of **1** was established as 3*β*-hydroxy-urs-30-*p*-*Z*-hydroxycinnamoyl-12-en-28-oic-acid.

1 R=*p*-*Z*-hydroxycinnamoyl
4 R=*p*-*E*-hydroxycinnamoyl

2

3

Compound **2** exhibited the same molecular formula $C_{39}H_{54}O_6$ as **1**, as established by HREIMS at *m*/*z* 618.3890 $[M]^+$. The NMR data of **2** (Table 1) were similar to those of oleanolic acid (**8**) except for the presence of one *p*-*E*-hydroxycinnamoyl group in the downfield region of **2** and the

*To whom correspondence should be addressed. E-mail: nhtan@mail.kib.ac.cn

**Table 1.** $^{1}$**H NMR and** $^{13}$**C NMR data for compounds 1–3 in pyridine-*d*$_5$**

| pos. | **1** $\delta_H$[d] (*J* in Hz) | **1** $\delta_C$[a], type | **2** $\delta_H$[d] (*J* in Hz) | **2** $\delta_C$[b], type | **3** $\delta_H$[c] (*J* in Hz) | **3** $\delta_C$[a], type |
|---|---|---|---|---|---|---|
| 1a | 0.98, overlap | 39.5, CH$_2$ | 1.01, overlap | 39.0, CH$_2$ | 1.09, overlap | 38.4, CH$_2$ |
| 1b | 1.56, overlap | | 1.54, m | | | |
| 2a | 1.85, overlap | 28.6, CH$_2$ | 1.85, m | 28.1, CH$_2$ | 1.92, m | 28.2, CH$_2$ |
| 2b | 2.34, m | | 2.18, m | | | |
| 3 | 3.48, dd (10.0, 6.0) | 78.6, CH | 3.46, m | 78.1, CH | 3.57, m | 78.8, CH |
| 4 | | 39.9, C | | 39.4, C | | 40.6, C |
| 5 | 0.86, overlap | 56.3, CH | 0.87, overlap | 55.8, CH | 1.34, d (10.6) | 61.4, CH |
| 6a | 1.37, overlap | 19.3, CH$_2$ | 1.37, m | 18.8, CH$_2$ | 4.43, dt (10.6, 3.6) | 68.0, CH |
| 6b | 1.56, overlap | | 1.56, overlap | | | |
| 7a | 1.37, overlap | 34.0, CH$_2$ | 1.32, overlap | 33.3, CH$_2$ | 1.96, overlap | 53.1, CH$_2$ |
| 7b | 1.56, overlap | | 1.49, m | | 2.58, dd (12.0, 3.6) | |
| 8 | | 40.4, C | | 39.8, C | | 42.1, C |
| 9 | 1.64, overlap | 48.5, CH | 1.67, overlap | 48.1, CH | 2.25, overlap | 51.2, CH |
| 10 | | 37.8, C | | 37.4, C | | 40.1, C |
| 11a | 1.97, overlap | 24.1, CH$_2$ | 1.94, m | 23.8, CH$_2$ | 2.48, dd (16.8,11.2) | 38.2, CH$_2$ |
| 11b | | | | | 2.80, dd (16.8, 9.2) | |
| 12 | 5.51, br. s | 126.7, CH | 5.56, br. s | 123.1, CH | | 215.8, C |
| 13 | | 139.3, C | | 139.9, C | | 54.2, C |
| 14 | | 42.9, C | | 42.2, C | | 157.2, C |
| 15 | 2.33, m | 29.1, CH$_2$ | 2.20, m | 28.3, CH$_2$ | 5.75, dd (8.0, 2.0) | 119.1, CH |
| 16a | 2.05, overlap | 25.3, CH$_2$ | 2.01, m | 23.9, CH$_2$ | 1.52, overlap | 38.1, CH$_2$ |
| 16b | 2.14, m | | 2.18, m | | 2.13, d (14.8) | |
| 17 | | 48.3, C | | 47.0, C | | 35.5, C |
| 18 | 2.69, d (11.0) | 53.8, CH | 3.40, m | 41.1, CH | 2.25, overlap | 49.3, CH |
| 19 | 1.85, overlap | 34.9, CH | 2.06, m | 41.1, CH$_2$ | 1.43, overlap | 37.6, CH |
| 20 | 1.42, overlap | 44.4, CH | | 35.2, C | 1.30, overlap | 37.4, CH |
| 21a | 1.62, overlap | 26.1, CH$_2$ | 1.37, m | 29.2, CH$_2$ | 1.05, overlap | 29.3, CH$_2$ |
| 21b | 1.85, overlap | | 1.69, m | | 1.60, m | |
| 22a | 1.97, overlap | 37.4, CH$_2$ | 1.90, m | 32.3, CH$_2$ | 1.25, overlap | 38.7, CH$_2$ |
| 22b | 2.05, overlap | | 2.09, m | | | |
| 23 | 1.26, s | 29.3, CH$_3$ | 1.25, s | 28.8, CH$_3$ | 2.02, s | 32.4, CH$_3$ |
| 24 | 1.04, s | 17.1, CH$_3$ | 1.05, s | 16.6, CH$_3$ | 1.48, s | 17.1, CH$_3$ |
| 25 | 0.90, s | 16.2, CH$_3$ | 0.91, s | 15.6, CH$_3$ | 1.03, s | 17.1, CH$_3$ |
| 26 | 1.07, s | 17.9, CH$_3$ | 1.05, s | 17.5, CH$_3$ | 1.07, s | 25.5, CH$_3$ |
| 27 | 1.22, s | 24.4, CH$_3$ | 1.32, s | 26.2, CH$_3$ | 1.43, s | 21.1, CH$_3$ |
| 28 | | 180.3, C | | 179.9, C | 0.88, s | 34.0, CH$_3$ |
| 29 | 1.07, overlap | 17.6, CH$_3$ | 1.19, s | 19.5, CH$_3$ | 1.14, d (6.4) | 25.1, CH$_3$ |
| 30a | 4.26, dd (11.0, 7.5) | 68.2, CH$_2$ | 4.14, d (10.5) | 74.9, CH$_2$ | 0.97, d (6.4) | 22.8, CH$_3$ |
| 30b | 4.49, dd (11.0, 3.0) | | 4.23, d (10.5) | | | |
| 1' | | 167.8, C | | 167.7, C | | |
| 2' | 6.07, d (13.0) | 116.9, CH | 6.77, d (16.0) | 115.3, CH | | |
| 3' | 7.01, d (13.0) | 144.6, CH | 8.07, d (16.0) | 144.6, CH | | |
| 4' | | 127.1, C | | 126.3, C | | |
| 5' | 8.10, d (8.5) | 134.1, CH | 7.68, d (8.0) | 130.8, CH | | |
| 6' | 7.22, overlap | 116.5, CH | 7.21, overlap | 116.9, CH | | |
| 7' | | 161.1, C | | 161.7, C | | |
| 8' | 7.22, overlap | 116.5, CH | 7.21, overlap | 116.9, CH | | |
| 9' | 8.10, d (8.5) | 134.1, CH | 7.68, d (8.0) | 130.8, CH | | |

[a]Date were measured at 100 MHz; [b]Date were measured at 125 MHz; [c]Date were measured at 400 MHz; [d]Date were measured at 500 MHz.

replacement of one methyl in **8** by one hydroxymethyl group ($\delta_C$ 74.9). HMBC correlations of H-30 with the ester carbonyl carbon ($\delta_C$ 167.7), C-19, C-20, C-21, and Me-29 indicated that the *p*-*E*-hydroxycinnamoyl group located at C-30 (Figure 1). The observed NOE correlations (Figure 2) of H-3/H-5 and Me-23, H-5/H-9, and H-9/Me-27 indicated that H-3, H-5, H-9, Me-23 and Me-27 are cofacial and assigned as $\alpha$-oriented. In turn the cross-peaks of Me-25/Me-24 and Me-26, and H-18/Me-29 indicated the $\beta$-oriented of H-18, Me-24, Me-25, Me-26 and Me-29. Thus, the structure of **2** was assigned as 3$\beta$-hydroxy-olean-30-*p*-*E*-hydroxycinnamoyl-12-en-28-oic-acid.

Compound **3** gave the molecular formula $C_{30}H_{48}O_3$, based on HRESIMS (*m/z* 479.3504 [M + Na]$^+$), requiring seven degrees of unsaturation. The IR spectrum showed absorption bands for hydroxyl (3442 cm$^{-1}$), carbonyl (1705 cm$^{-1}$) and olefinic (1640 cm$^{-1}$) groups. The $^{13}$C NMR spectrum data (Table 1) showed the presence of 30 carbon signals due to one trisubstituted double bond ($\delta_C$ 119.1, 157.2), one ketone carbon ($\delta_C$ 215.8), eight methyls, seven methylenes, seven

**Figure 1.** Key $^{1}$H-$^{1}$H COSY and HMBC correlations of **1–3**

methines (two oxygenated), and five quaternary carbons. Comparison of the NMR data of **3** with those of ursolic acid suggested that their structures are closely related.[10] The main differences were that one characteristic trisubstituted double bond at C-12/C-13 in conventional ursane-type triterpenoids was absent in **3**, while one different trisubstituted double bond, one carbonyl group, and one additional hydroxy group were present. The double bond was placed between C-14 and C-15, as determined by HMBC correlations (Figure 1) of H-15 ($\delta_H$ 5.75) with C-17 ($\delta_C$ 35.5) and of Me-26 and Me-27 with C-14 ($\delta_C$ 157.2). The location of the ketone carbon ($\delta_C$ 215.8) at C-12 was elucidated by HMBC correlations of H-9, H-11 and Me-27 with C-12. In addition, the position of the additional hydroxy group at C-6 was deduced by correlations of H-6 with H-5 in the $^1$H-$^1$H COSY spectrum combined with HMBC correlations of H-5 with C-6 (Figure 1). Thus, the planar structure of **3** was established. The $\alpha$-orientations of H-3, H-5, H-9, and Me-23 were established by NOE correlations of H-3/H-5 and Me-23 and H-5/H-9, and the $\beta$-orientations of H-6, H-18, H-20, Me-24, Me-25, Me-26, Me-27, Me-28, Me-29 were deduced by NOE correlations of H-6/Me-24 and Me-25, H-7$\beta$/H-6 and Me-26, H-18/Me-27, Me-28 and Me-29, and Me-29/H-20 (Figure 2). Accordingly, the structure of **3** was elucidated as 3$\beta$, 6$\alpha$-dihydroxy-urs-14-en-12-one.

**Figure 2.** Key ROESY correlations of **1**–**3**

All compounds were evaluated for cytotoxicity against three human cancer cell lines, Hela (human cervical carcinoma), BGC-823 (human stomach adenocarcinoma), and A549 (human lung adenocarcinoma), and results indicated that compounds **2**–**6** showed cytotoxicity with the $IC_{50}$ values of 10.75~18.87 $\mu$g/mL (Table 2).

**Table 2. Cytotoxicity of 1–10 against cancer cell lines[a] with $IC_{50}$ ($\mu$g/mL)**

| compound | Hela | A549 | BGC-823 |
|---|---|---|---|
| **1** | > 20 | > 20 | > 20 |
| **2** | 16.18 | >20 | > 20 |
| **3** | > 20 | 15.74 | > 20 |
| **4** | 13.53 | > 20 | 14.18 |
| **5** | 12.39 | 18.87 | 10.75 |
| **6** | 11.80 | >20 | 11.89 |
| **7** | > 20 | > 20 | > 20 |
| **8** | > 20 | > 20 | > 20 |
| **9** | > 20 | > 20 | > 20 |
| **10** | > 20 | > 20 | > 20 |
| Taxol[b] | 0.38 | 0.02 | 0.01 |

[a]Cell lines: Hela human cervical carcinoma; BGC-823 human stomach adenocarcinoma; A549 human lung adenocarcinoma.
[b]positive control.

## Experimental Section

**General Experimental Procedures.** Optical rotations were measured with a Horiba SEPA-300 polarimeter. IR spectra were obtained by a Bruker FT-IR Tensor 27 spectrophotometer using KBr pellets. UV spectra were obtained using a Shimadzu UV-2401A spectrophotometer. 1D and 2D NMR spectra were recorded on Bruker AX-400, DRX-500, or AV-600 spectrometers with TMS as an internal standard. Chemical shifts ($\delta$) were expresses in ppm with reference to solvent signals. HREIMS were recorded on a Waters Auto Premier P776 spectrometer. HRESIMS were recorded on an API QSTER time-of-flight spectrometer. Analytical or Semipreparative HPLC was performed on an Agilent 1100 liquid chromatograph with a Zorbax Eclipse-$C_{18}$ (4.6 mm × 150 mm; 9.4 mm × 250 mm) column. Cloumn chromatographies were performed using silica gel (200–300 mesh, Qingdao Yu-Ming-Yuan Chemical Co. Ltd., Qingdao, China), Sephadex LH-20 (Pharmacia Fine Chemical Co., Uppsala, Sweden), and Lichroprep RP-18 gel (40–63 $\mu$M, Merck, Darmstadt, Germany). Fractions were monitored by TLC (GF 254, Qingdao Yu-Ming-Yuan Chemical Co. Ltd., Qingdao, China), and spots were visualized by heating silica gel plates sprayed with 10% $H_2SO_4$ in EtOH.

**Plant Materal.** The roots of *R. schumanniana* were purchased in August 2009 from the Yunnan Lv-Sheng Pharmaceutical Co. Ltd., Kunming, China. The material was identified by Prof. Xi-Wen Li of Kunming Institute of Botany. A voucher specimen (KUN0328859) was deposited at the Herbarium of Kunming Institute of Botany, Chinese Academy of Sciences.

**Extraction and Isolation.** The dried and powdered roots of *R. schumanniana* (50 kg) were extracted with 70% aqueous MeOH (40 L × 3) for 12 hours at room temperature. After removal of the solvent under reduced pressure, the MeOH extract (18.6 kg) was suspended in $H_2O$ and partitioned successively with EtOAc and *n*-BuOH to give an EtOAc-soluble portion (3.7 kg) and a *n*-BuOH-soluble portion (4.2 kg). The EtOAc part was chromatographed on silica gel column eluting with chloroform-methanol (1:0, 95:5, 9:1, 8:2, 7:3, and 0:1) to afford fractions I–IV. Fraction I (9:1, 163 g) was further chromatographed on silica gel using a petroleum ether-acetone gradient (10:1 to 0:1) as the eluent to yield 6 subfractions, I-1–I-6. Subfractions I-1 was chromatographed with RP-18, and then separated by semi-preparative HPLC ($CH_3CN$:$H_2O$, 80:20) to yield **1** (12 mg) and **2** (2.6 mg). **4** (23 mg) was purified from subfractions I-2 by repeated chromatographed with silica gel. Subfractions I-4 was chromatographed on silica gel using a chloroform-methanol gradient (50:1 to 10:1) as the eluent, and then purified over Sephadex LH-20 eluted with chloroform-methanol (1:1), then by semi-preparative HPLC ($CH_3CN$:$H_2O$, 60:40 and 73:27) to yield **5** (22 mg), **6** (8 mg), **8** (35 mg) and **3** (7 mg), respectively. Subfractions I-6 was chromatographed on silica gel using a chloroform-acetone gradient (50:1 to 0:1) as the eluent, and then purified by semi-preparative HPLC ($CH_3CN$:$H_2O$, 65:35) to yield **7** (15 mg), **9** (10.2 mg) and **10** (6 mg).

**3*β*-Hydroxy-urs-30-*p*-*Z*-hydroxycinnamoyl-12-en-28-oic-acid (1):** white powder; $[\alpha]_D^{16}$ + 6.5 (*c* 0.08, MeOH:$CHCl_3$ = 1:1); UV (MeOH) $\lambda_{max}$ (log ε) 202 (4.16), 312 (4.14) nm; IR (KBr) $\nu_{max}$ 3426, 2965, 2937, 2873, 1689, 1632, 1606, 1514, 1456, 1377, 1311, 1277, 1258, 1202, 1184, 1029, 997, 833, 519 $cm^{-1}$; $^1H$ and $^{13}C$ NMR data, see Table 1; negative ESIMS *m/z* 617 $[M - H]^-$; negative HRESIMS *m/z* 617.3856 $[M - H]^-$ (calcd for $C_{39}H_{53}O_6$, 617.3842).

**3*β*-Hydroxy-olean-30-*p*-*E*-hydroxycinnamoyl-12-en-28-oic-acid (2):** white amorphous powder; $[\alpha]_D^{16}$ + 13.1 (*c* 0.13, MeOH); UV (MeOH) $\lambda_{max}$ (log ε) 202 (4.16), 227 (4.01), 313 (4.22) nm; IR (KBr) $\nu_{max}$ 3429, 2938, 2875, 1692, 1632, 1606, 1515, 1456, 1387, 1167, 1027, 996, 833, 519 $cm^{-1}$; $^1H$ and $^{13}C$ NMR data, see Table 1; positive EIMS *m/z* 618 $[M]^+$; positive HREIMS *m/z* 618.3890 $[M]^+$ (calcd for $C_{39}H_{54}O_6$, 618.3920).

**3*β*, 6*α*-Dihydroxy-urs-14-en-12-one (3):** white amorphous powder; $[\alpha]_D^{20}$ – 10.7 (*c* 0.10, MeOH); UV (MeOH) $\lambda_{max}$ (log ε) 201 (3.41) nm; IR (KBr) $\nu_{max}$ 3442, 2927, 2866, 1705, 1640, 1462, 1382, 1140, 1036, 987 $cm^{-1}$; $^1H$ and $^{13}C$ NMR data, see Table 1; positive ESIMS *m/z* 479 $[M + Na]^+$; positive HRESIMS *m/z* 479.3504 $[M + Na]^+$ (calcd for $C_{30}H_{48}O_3Na$, 479.3501).

**Cytotoxicity Assay.** The cytotoxicity of all compounds against Hela, A549, and BGC-823 cancer cell lines was measured using the sulforhodamine B (SRB) assay. Taxol was used as positive control. Cells were plated in 96-well culture plates for 24h before treated with serial dilutions of all compounds. After being incubated for 48 h, cells were fixed with 25 $\mu$L of ice-cold 50% trichloroacetic acid and incubated at 4 $^O$C for 1 h. After washing with distilled water and air-drying, the plate was stained for 15 min with 100 $\mu$L of 0.4% SRB (Sigma) in 1% glacial acetic acid. The plates were washed with 1% acetic acid and air-dried. For reading the plate, the protein-bound dye was dissolved in 100 $\mu$L of 10 mM Tris base. The absorbance was measured at 560 nm. All tests were performed in triplicate, and results are expressed as $IC_{50}$ values.

## Electronic Supplementary Material

Supplementary material is available in the online version of this article at http://dx.doi.org/ 10.1007/s13659-012-0038-8 and is accessible for authorized users.

## Acknowledgments

This work was supported by the National Natural Science Foundation of China (U1032602, 91013002, 30725048), the National New Drug Innovation Major Project of China (2011ZX09307-002-02), and the National Basic Reaearch Program of China (2009 CB522300).

**Open Access** This article is distributed under the terms of the Creative Commons Attribution License which permits any use, distribution, and reproduction in any medium, provided the original author(s) and source are credited.

## Electronic supplementary material

Supplementary material, approximately 3.36 MB.

## References

[1] Liu Y. L., Bai Y. L. (1985). Acta Pharm. Sin..

[2] Liu Y. L., Chen B. Z., Bai Y. L., Duddeck H., Hiegemann M. (1991). Phytochemistry.

[3] Siddiqui B. S., Firdous, Begum S. (1999). Phytochemistry.

[4] Wu Y. X., Zhang W., Li J. C., Yang L. J., Liu N. (2011). Chin. Tradit. Herb. Drugs.

[5] Zhu C. C., Gao L., Zhao Z. X., Lin C. Z. (2012). Acta Pharm. Sin..

[6] Talapatra S. K., Sarkar A. C., Talapatra B. (1981). Phytochemistry.

[7] Sun W. (2012). Chin. Tradit. Herb. Drugs.

[8] Khan M. A., Atta-ur-Rahman (1975). Phytochemistry.

[9] Zou C., Hao X. J., Chen C. X., Zhou J. (1992). Acta Bot. Yunnan..

[10] Seebacher W., Simic N., Weis R., Saf R., Kunert O. (2003). Magn. Reson. Chem..

