# Supplementary material for: Three new triterpenoids from Rubia schumanniana
Source: Nat Prod Bioprospect. 2012 Jul 12;2(4):166–9. doi: 10.1007/s13659-012-0038-8 (PMC4131627; doi:10.1007/s13659-012-0038-8)
Supplement: Supplementary file 1 — Supplementary material, approximately 3.36 MB. [file 13659_2012_38_MOESM1_ESM.pdf]

Electronic Supplementary Material

Three new triterpenoids from *Rubia schumanniana*

Bin KUANG,[a,b] Jing HAN,[a,b] Guang-Zhi ZENG,[a] Xiao-Qiang CHEN,[a] Wen-Jun HE,[a] and Ning-Hua TAN[a,]*

[a]State Key Laboratory of Phytochemistry and Plant Resources in West China, Kunming Institute of Botany, Chinese Academy of Sciences, Kunming 650201, China

[b]Graduate University of Chinese Academy of Sciences, Beijing 100049, China

Received 15 May 2012; Accepted 25 June 2012

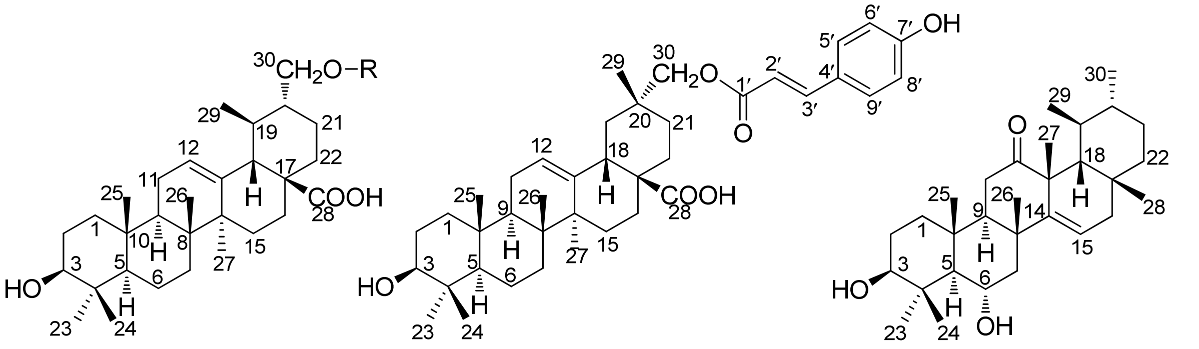

1 R=*p*-*Z*-hydroxycinnamoyl

4 R=*p*-*E*-hydroxycinnamoyl

2

3

Structures of compounds **1**–**4**

*To whom correspondence should be addressed. E-mail: nhtan@mail.kib.ac.cn

Contents

**S1**	The ^{1}H NMR spectrum of 3*β*-hydroxy-urs-30-*p*-*Z*-hydroxycinnamoyl-12-en-28-oic-acid (**1**) in C_5D_5N
**S2**	The ^{13}C NMR spectrum of 3*β*-hydroxy-urs-30-*p*-*Z*-hydroxycinnamoyl-12-en-28-oic-acid (**1**) in C_5D_5N
**S3**	The HSQC spectrum of 3*β*-hydroxy-urs-30-*p*-*Z*-hydroxycinnamoyl-12-en-28-oic-acid (**1**)
**S4**	The ^{1}H-^{1}H COSY spectrum of 3*β*-hydroxy-urs-30-*p*-*Z*-hydroxycinnamoyl-12-en-28-oic-acid (**1**)
**S5**	The HMBC spectrum of 3*β*-hydroxy-urs-30-*p*-*Z*-hydroxycinnamoyl-12-en-28-oic-acid (**1**)
**S6**	The ROESY spectrum of 3*β*-hydroxy-urs-30-*p*-*Z*-hydroxycinnamoyl-12-en-28-oic-acid (**1**)
**S7-S11**	The MS, IR, UV, $[\alpha]_D$ spectrums of 3*β*-hydroxy-urs-30-*p*-*Z*-hydroxycinnamoyl-12-en-28-oic-acid (**1**)
**S12**	The ^{1}H NMR spectrum of 3*β*-hydroxy-olean-30-*p*-*E*-hydroxycinnamoyl-12-en-28-oic-acid (**2**) in C_5D_5N
**S13**	The ^{13}C NMR spectrum of 3*β*-hydroxy-olean-30-*p*-*E*-hydroxycinnamoyl-12-en-28-oic-acid (**2**) in C_5D_5N
**S14**	The HSQC spectrum of 3*β*-hydroxy-olean-30-*p*-*E*-hydroxycinnamoyl-12-en-28-oic-acid (**2**)
**S15**	The ^{1}H-^{1}H COSY spectrum of 3*β*-hydroxy-olean-30-*p*-*E*-hydroxycinnamoyl-12-en-28-oic-acid (**2**)
**S16**	The HMBC spectrum of 3*β*-hydroxy-olean-30-*p*-*E*-hydroxycinnamoyl-12-en-28-oic-acid (**2**)
**S17**	The ROESY spectrum of 3*β*-hydroxy-olean-30-*p*-*E*-hydroxycinnamoyl-12-en-28-oic-acid (**2**)
**S18-S22**	The MS, IR, UV, $[\alpha]_D$ spectrums of 3*β*-hydroxy-olean-30-*p*-*E*-hydroxycinnamoyl-12-en-28-oic-acid (**2**)
**S23**	The ^{1}H NMR spectrum of 3*β*, 6*α*-dihydroxy-urs-14-en-12-one (**3**) in C_5D_5N
**S24**	The ^{13}C NMR spectrum of 3*β*, 6*α*-dihydroxy-urs-14-en-12-one (**3**) in C_5D_5N
**S25**	The HSQC spectrum of 3*β*, 6*α*-dihydroxy-urs-14-en-12-one (**3**)
**S26**	The ^{1}H-^{1}H COSY spectrum of 3*β*, 6*α*-dihydroxy-urs-14-en-12-one (**3**)
**S27**	The HMBC spectrum of 3*β*, 6*α*-dihydroxy-urs-14-en-12-one (**3**)
**S28**	The ROESY spectrum of 3*β*, 6*α*-dihydroxy-urs-14-en-12-one (**3**)
**S29-S33**	The MS, IR, UV, $[\alpha]_D$ spectrums of 3*β*, 6*α*-dihydroxy-urs-14-en-12-one (**3**)

S1 The ^{1}H NMR spectrum of 3*β*-hydroxy-urs-30-*p*-*Z*-hydroxycinnamoyl-12-en-28-oic-acid (1) in C_5D_5N

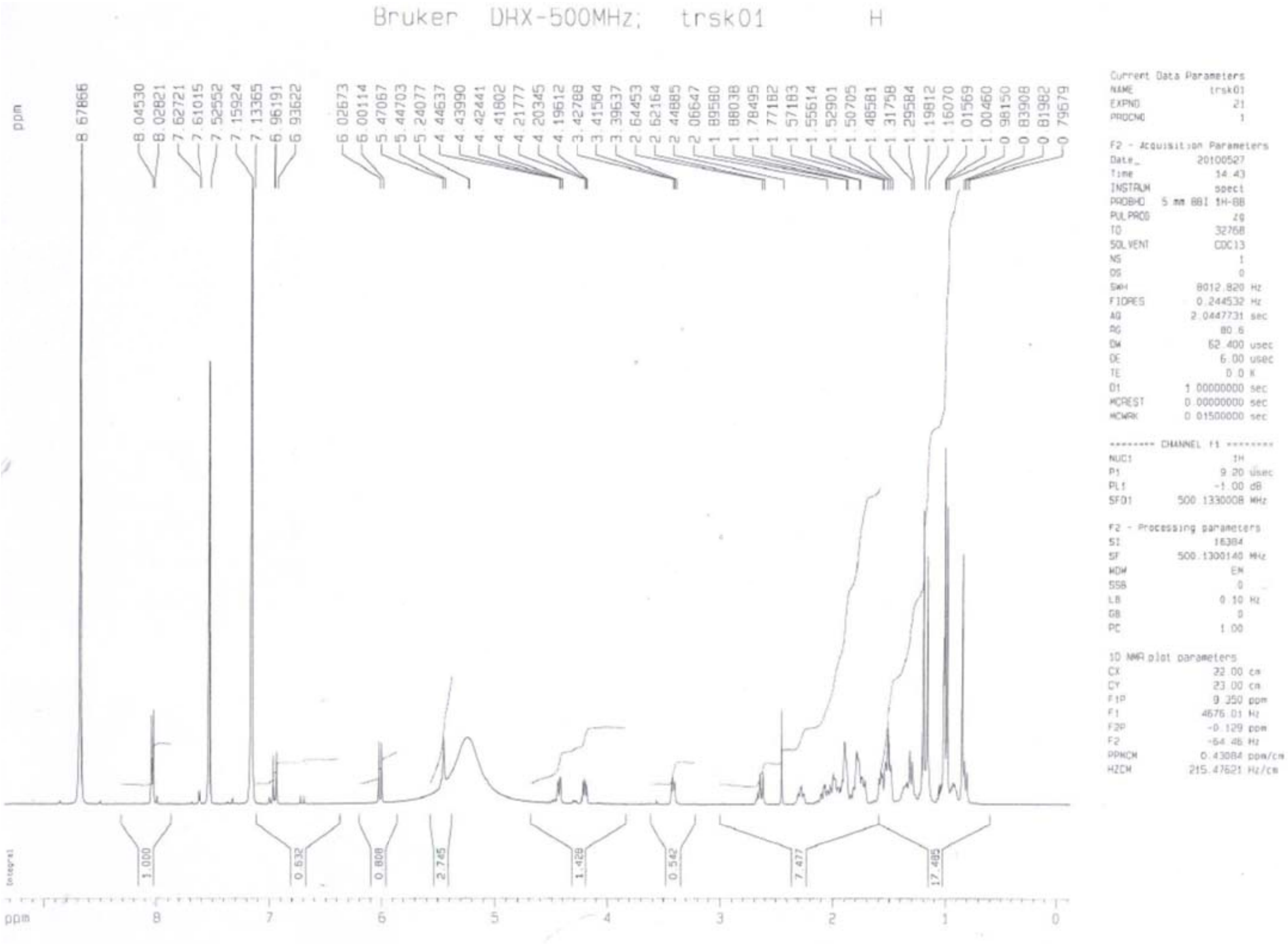

S2 The ^{13}C NMR spectrum of 3*β*-hydroxy-urs-30-*p*-*Z*-hydroxycinnamoyl-12-en-28-oic-acid (1) in C_5D_5N

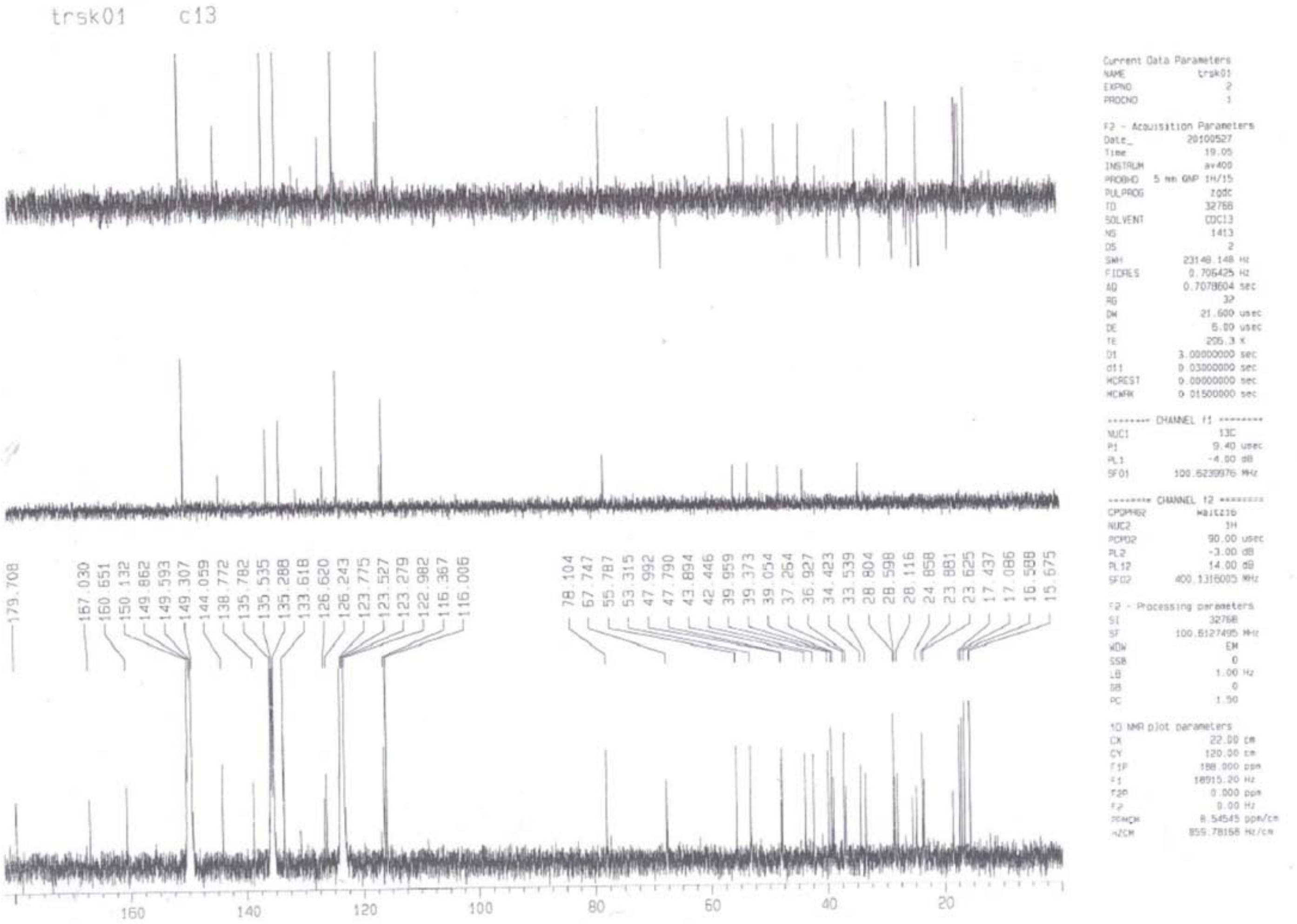

S3 The HSQC spectrum of 3*β*-hydroxy-urs-30-*p*-*Z*-hydroxycinnamoyl-12-en-28-oic-acid (1)

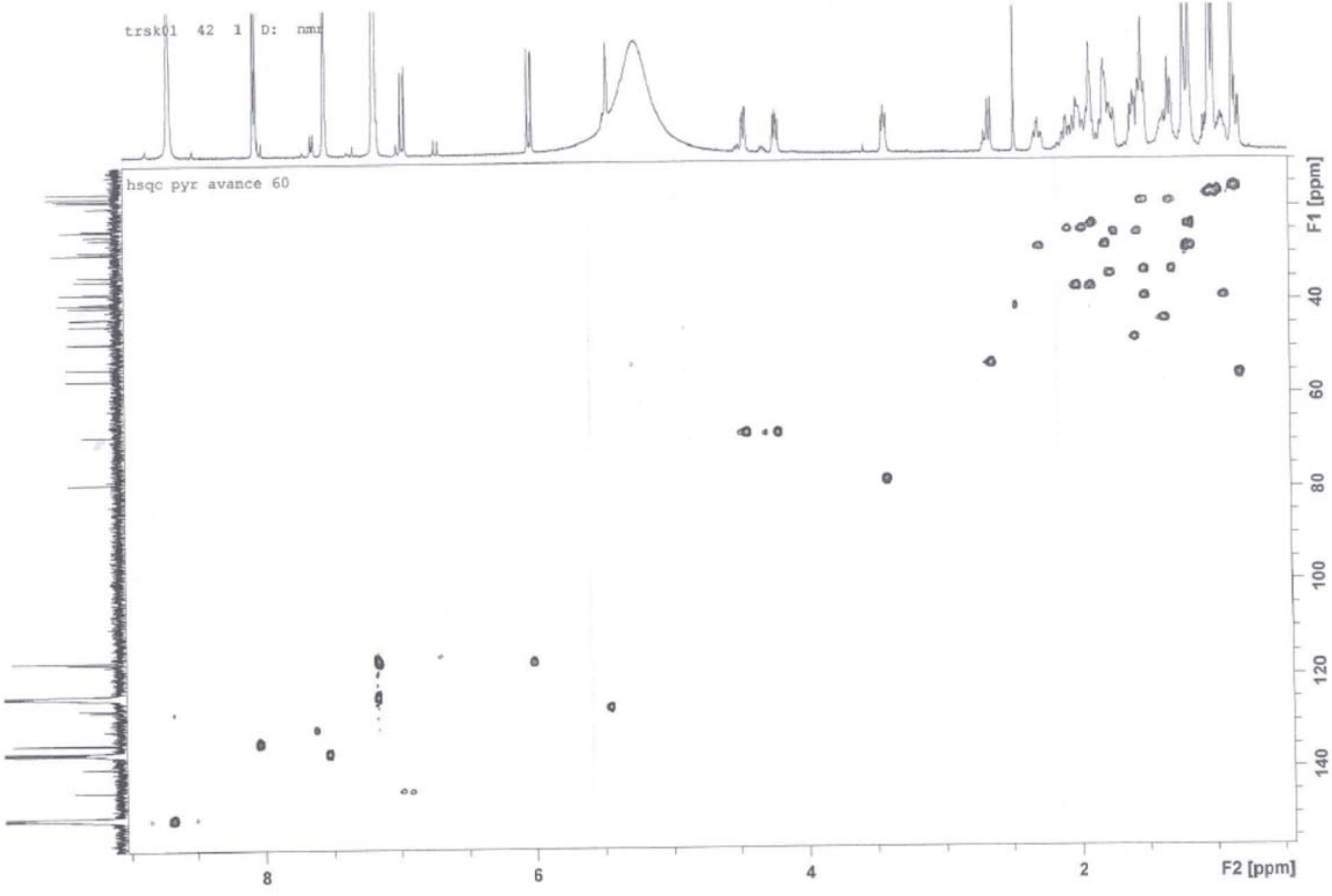

S4 The ^{1}H-^{1}H COSY spectrum of 3*β*-hydroxy-urs-30-*p*-*Z*-hydroxycinnamoyl-12-en-28-oic-acid (1)

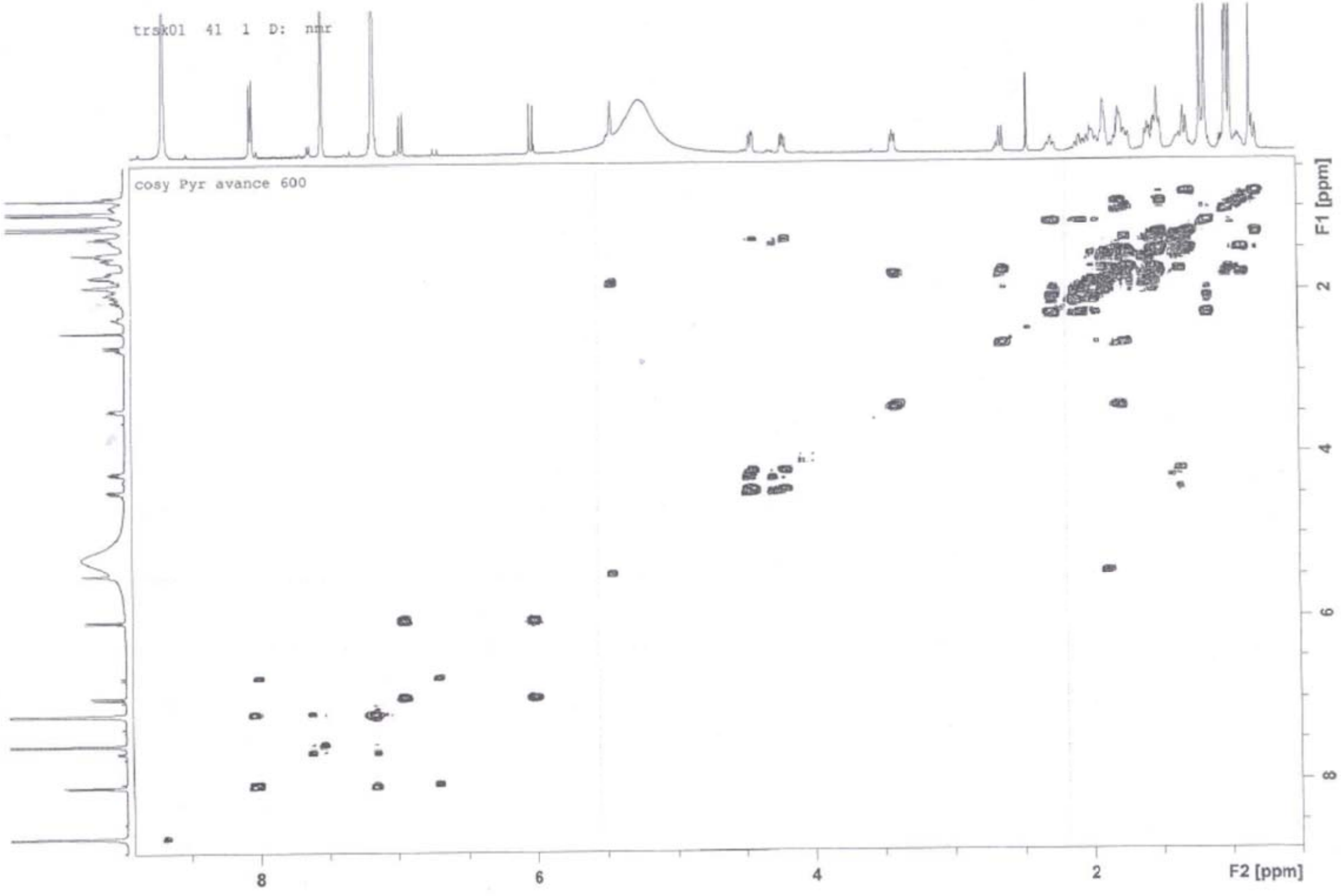

S5 The HMBC spectrum of 3*β*-hydroxy-urs-30-*p*-*Z*-hydroxycinnamoyl-12-en-28-oic-acid (1)

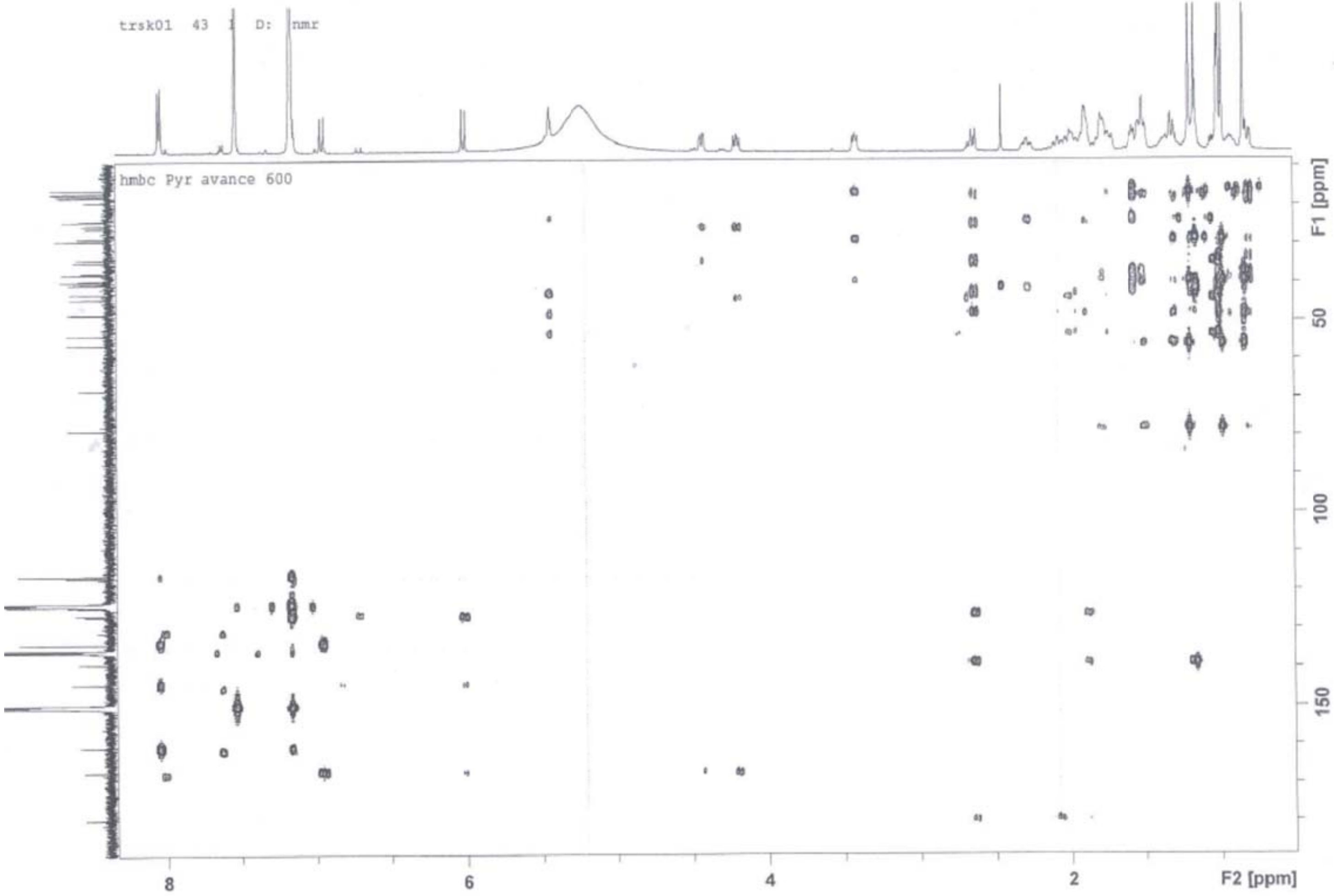

S6 The ROESY spectrum of 3*β*-hydroxy-urs-30-*p*-*Z*-hydroxycinnamoyl-12-en-28-oic-acid (1)

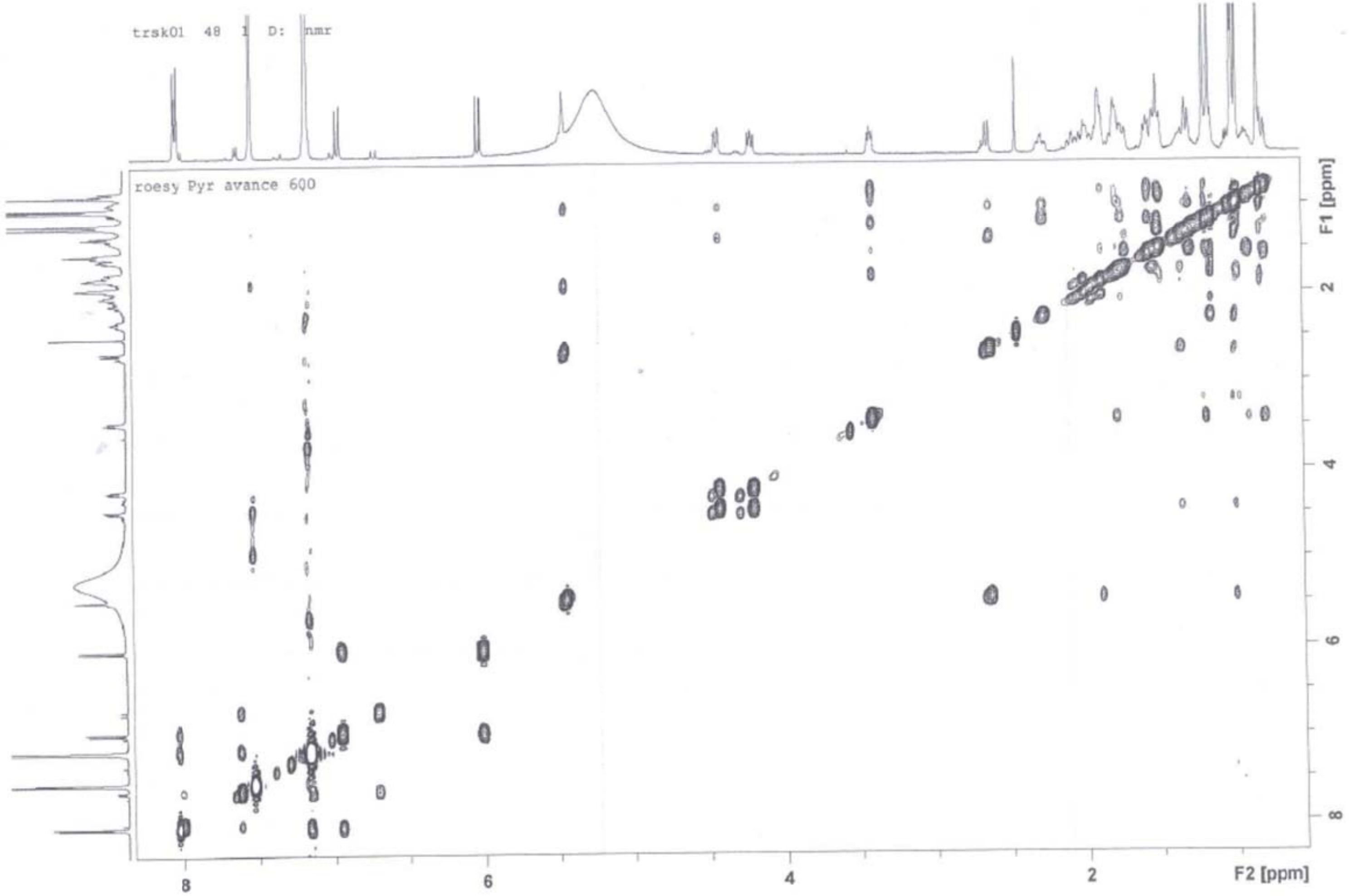

S7 The negative ESIMS spectrum of 3*β*-hydroxy-urs-30-*p*-*Z*-hydroxycinnamoyl-12-en-28-oic-acid (1)

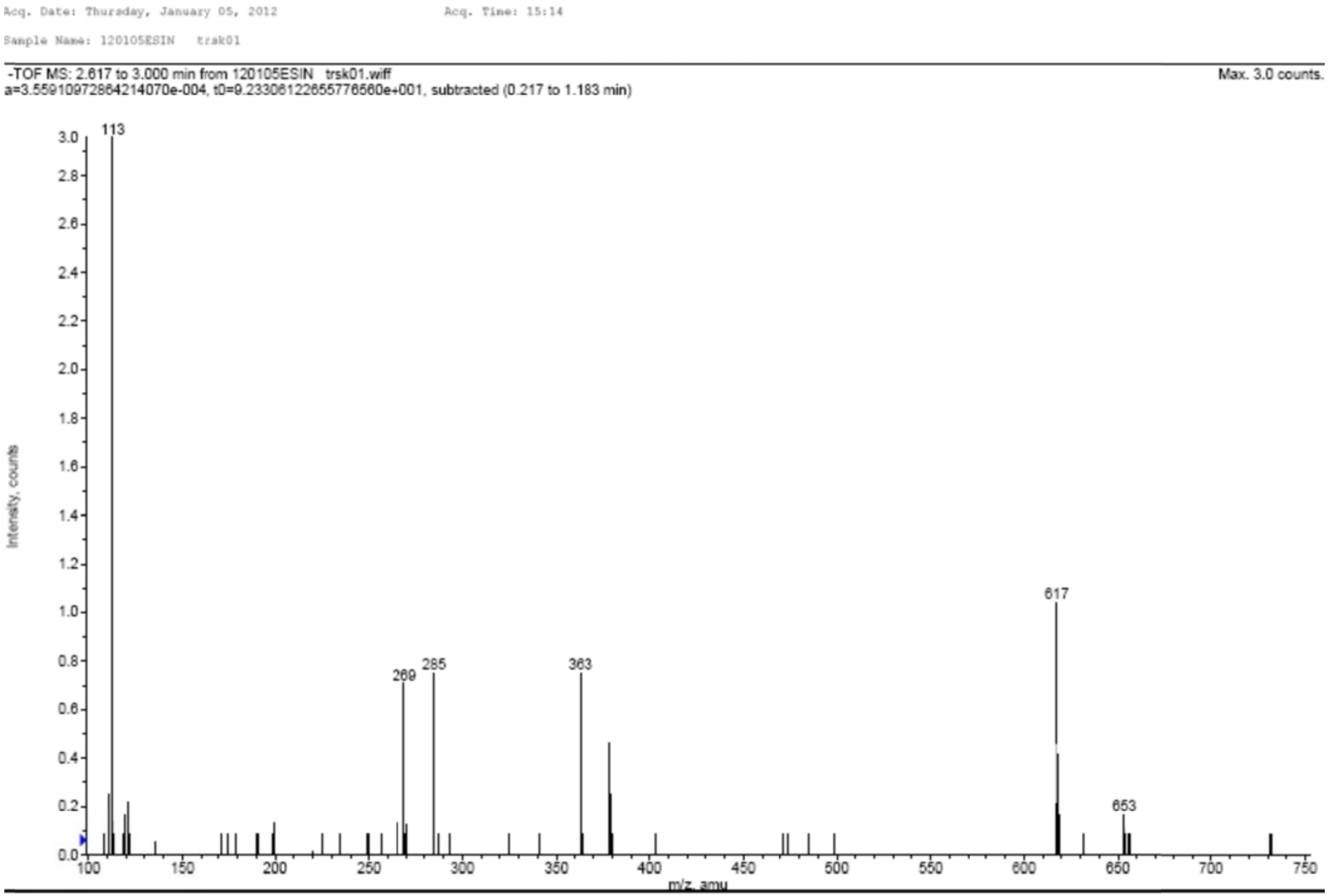

S8 a) The HRESIMS spectrum of 3*β*-hydroxy-urs-30-*p*-*Z*-hydroxycinnamoyl-12-en-28-oic-acid (1)

Acq. Date: Thursday, January 05, 2012 Acq. Time: 15:18

Sample Name: 120105ESINA trsk01

Elemental composition calculator

Target m/z: +617.3856 amu
Tolerance: +10.0000 ppm
Result type: Elemental
Max num of results: 1000
Min DBE: -10.0000 Max DBE: +60.0000
Electron state: OddAndEven
Num of charges: 0
Add water: N/A
Add proton: N/A
File Name: 120105ESINA trsk01.wiff

	Elements	Min Number	Max Number
1	2H	0	0
2	Br	0	0
3	C	0	200
4	Cl	0	0
5	F	0	0
6	H	0	400
7	I	0	0
8	K	0	0
9	N	0	0
10	Na	0	0
11	O	3	7

S8 b) The HRESIMS spectrum of 3*β*-hydroxy-urs-30-*p*-*Z*-hydroxycinnamoyl-12-en-28-oic-acid (1)

Acq. Date: Thursday, January 05, 2012 Acq. Time: 15:18

Sample Name: 120105ESINA trsk01

	Elements	Min Number	Max Number
12	P	0	0
13	Pt	0	0
14	S	0	0
15	Si	0	0

	Formula	Calculated m/z (amu)	mDa Error	PPM Error	DBE
1	C39 H53 O6	617.3842	1.3850	2.2433	13.5

S9 The IR spectrum of 3*β*-hydroxy-urs-30-*p*-*Z*-hydroxycinnamoyl-12-en-28-oic-acid (1)

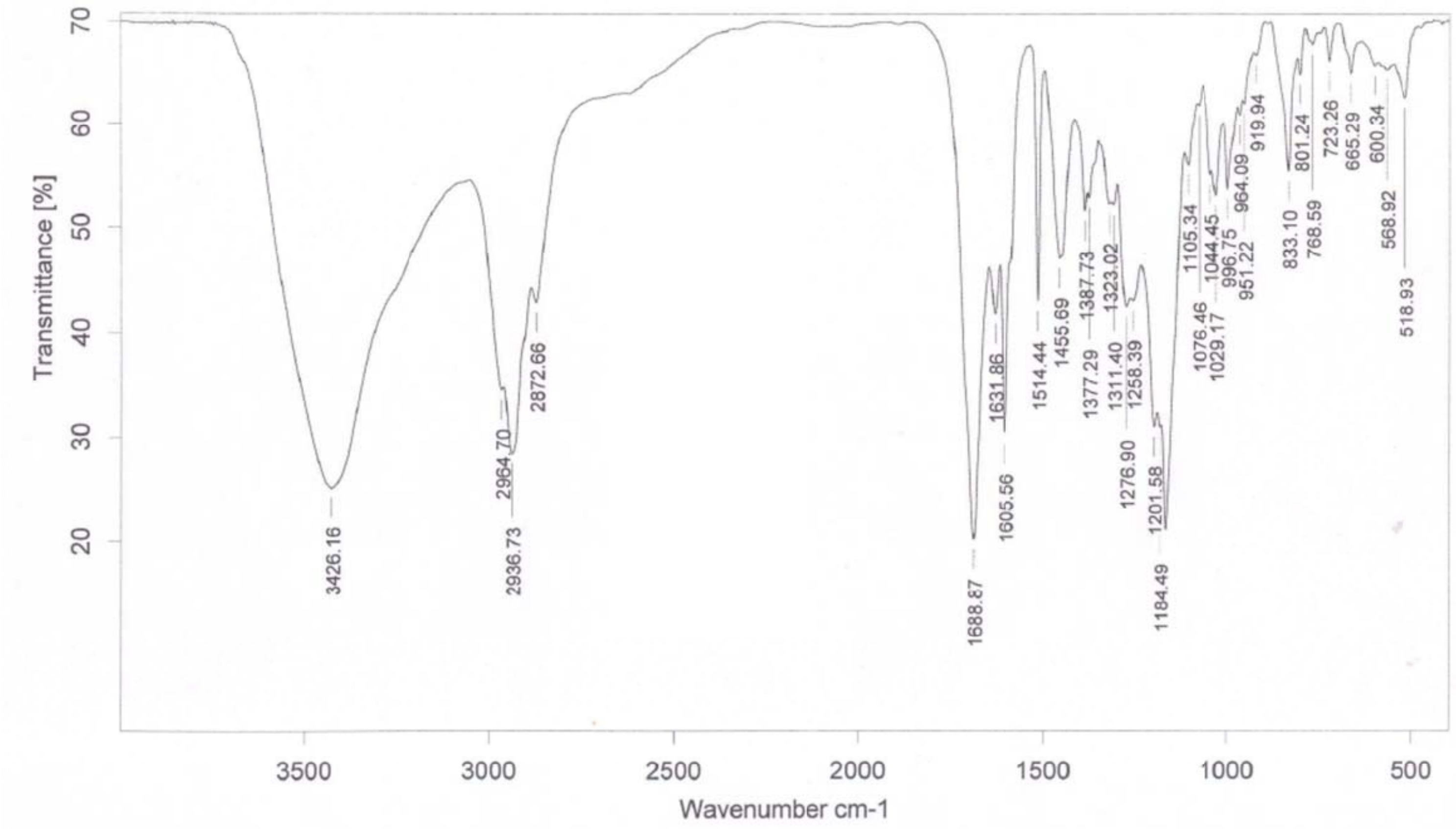

Sample : trsk01	Frequency Range : 399.246 - 3996.32		Measured on : 06/01/2012
Technique : KBr压片	Resolution : 4	Instrument : Tensor27	Sample Scans : 16
Customer : 120106IR0	Zerofilling : 2	Acquisition : Double Sided,For	

S10 The UV spectrum of 3*β*-hydroxy-urs-30-*p*-*Z*-hydroxycinnamoyl-12-en-28-oic-acid (1)

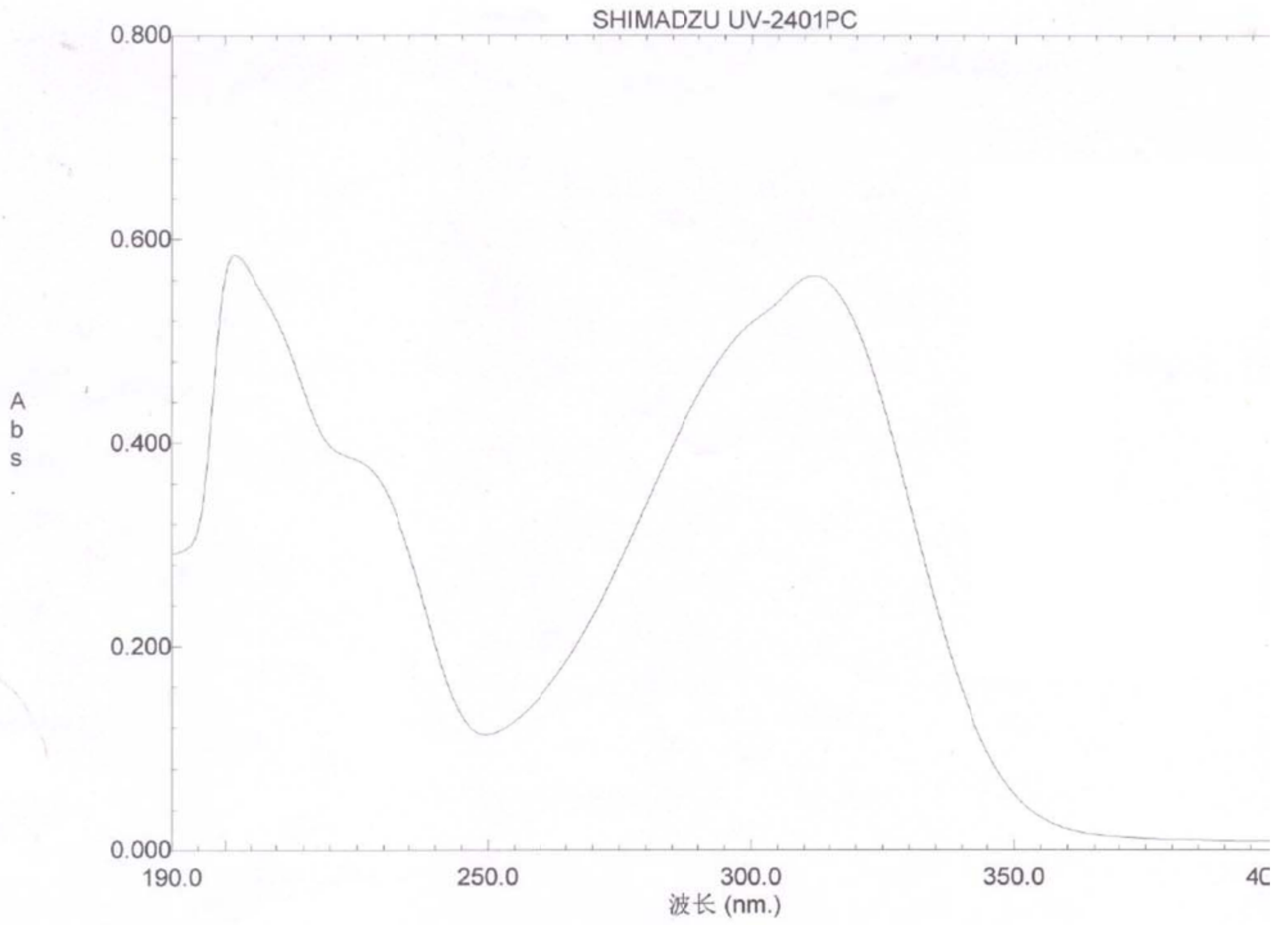

文件名: TRSK01

TRSK01 ———

样品浓度：0.0252毫克/毫升
溶剂：甲醇

创建于: 15:52 12-01-08
数据: 原始

测量模式: Abs.
扫描速度: 中速
狭缝: 5.0
采样间隔: 0.2

否.	波长 (nm.)	Abs.
1	311.60	0.5641
2	202.00	0.5846

S11 The $[\alpha]_D$ spectrum of 3*β*-hydroxy-urs-30-*p*-*Z*-hydroxycinnamoyl-12-en-28-oic-acid (1)

Optical rotation measurement

Model : P-1020 (A060460638)

No.	Sample	Mode	Data	Monitor<br>Blank	Temp.<br>Cell<br>Temp Point	Date<br>Comment<br>Sample Name	Light<br>Filter<br>Operator	Cycle Time<br>Integ Time
No.1	12 (1/3)	Sp.Rot	6.7500	0.0027<br>0.0000	15.5<br>50.00<br>Cell	Fri Jan 06 15:06:22 2012<br>0.00080g/mlMeOH+CHCl3(1:1)<br>TRSK01	Na<br>589nm	2 sec<br>10 sec
No.2	12 (2/3)	Sp.Rot	5.5000	0.0022<br>0.0000	15.5<br>50.00<br>Cell	Fri Jan 06 15:06:35 2012<br>0.00080g/mlMeOH+CHCl3(1:1)<br>TRSK01	Na<br>589nm	2 sec<br>10 sec
No.3	12 (3/3)	Sp.Rot	7.2500	0.0029<br>0.0000	15.5<br>50.00<br>Cell	Fri Jan 06 15:06:49 2012<br>0.00080g/mlMeOH+CHCl3(1:1)<br>TRSK01	Na<br>589nm	2 sec<br>10 sec

+6.5000°

S12 The ^{1}H NMR spectrum of 3*β*-hydroxy-olean-30-*p*-*E*-hydroxycinnamoyl-12-en-28-oic-acid (2) in C_5D_5N

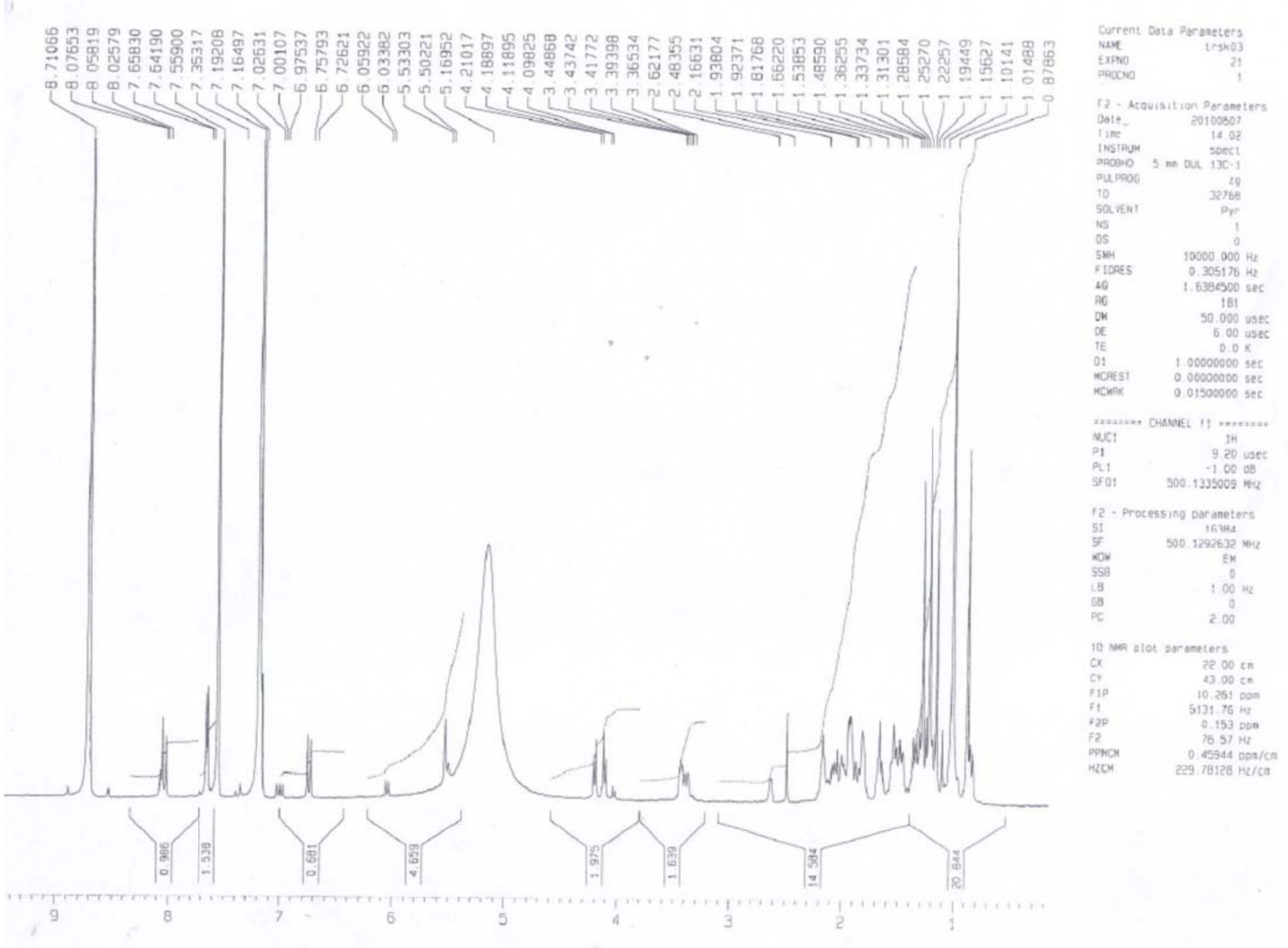

S13 The ^{13}C NMR spectrum of 3*β*-hydroxy-olean-30-*p*-*E*-hydroxycinnamoyl-12-en-28-oic-acid (2) in C_5D_5N

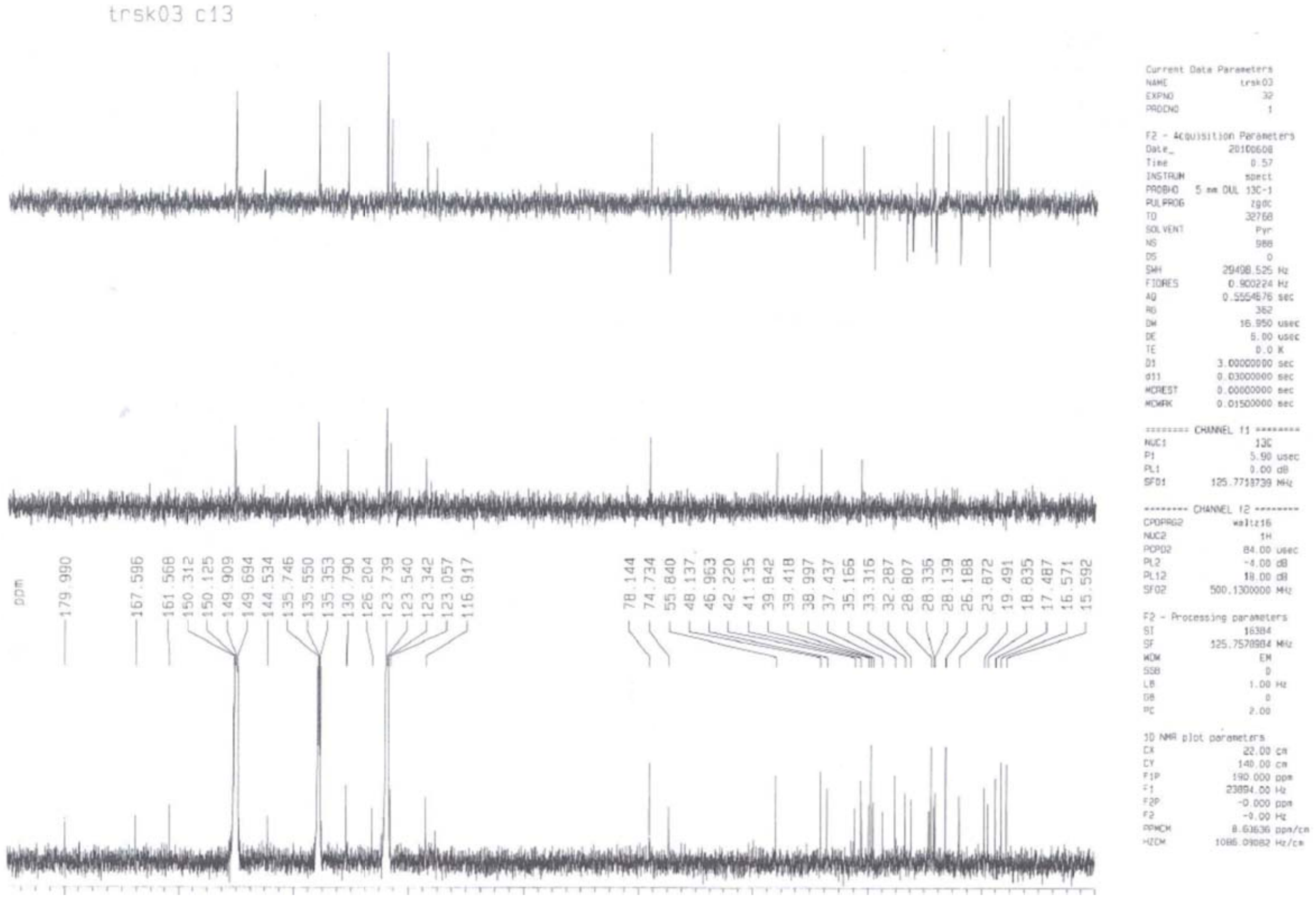

S14 The HSQC spectrum of 3*β*-hydroxy-olean-30-*p*-*E*-hydroxycinnamoyl-12-en-28-oic-acid (2)

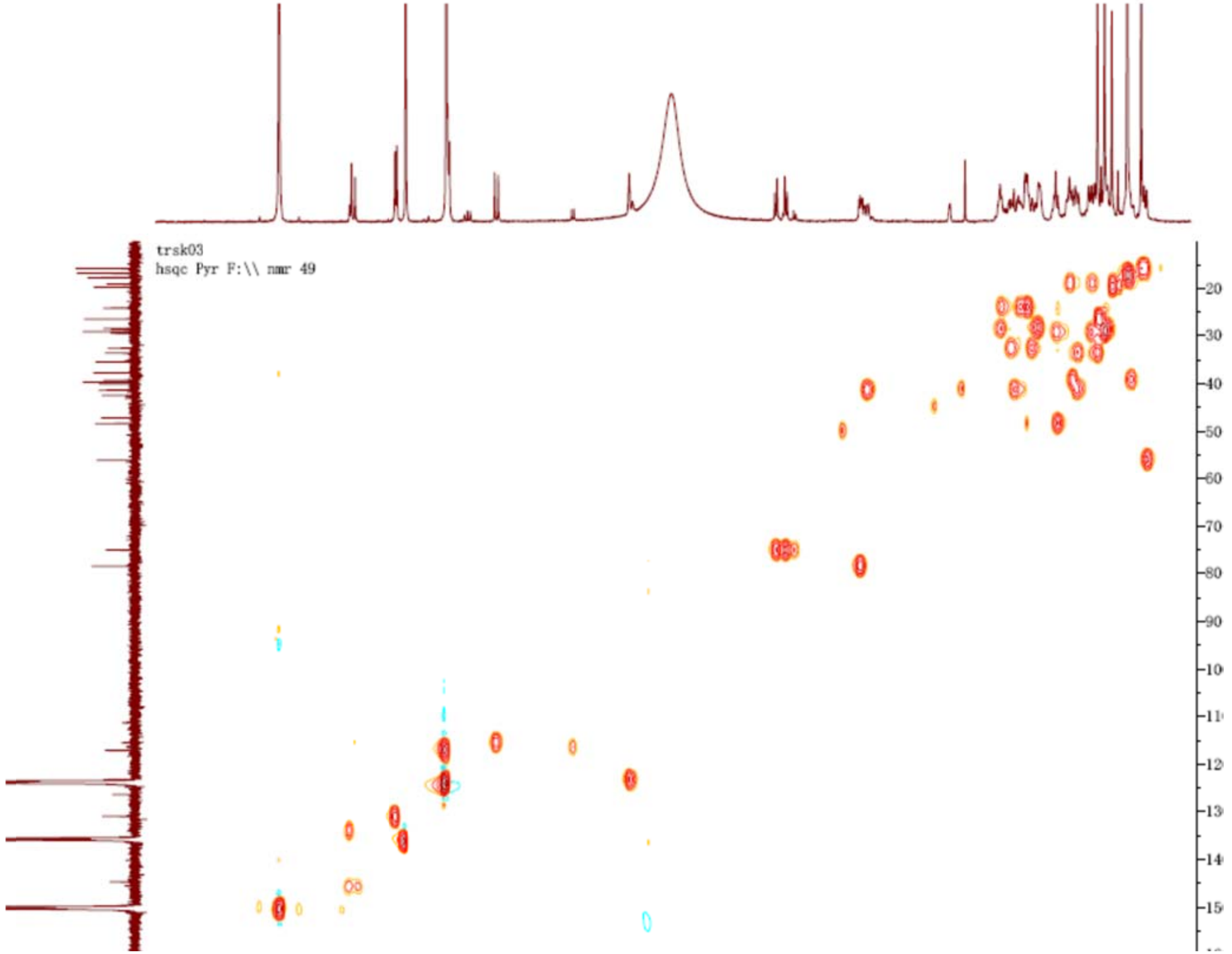

S15 The ^{1}H-^{1}H COSY spectrum of 3*β*-hydroxy-olean-30-*p*-*E*-hydroxycinnamoyl-12-en-28-oic-acid (2)

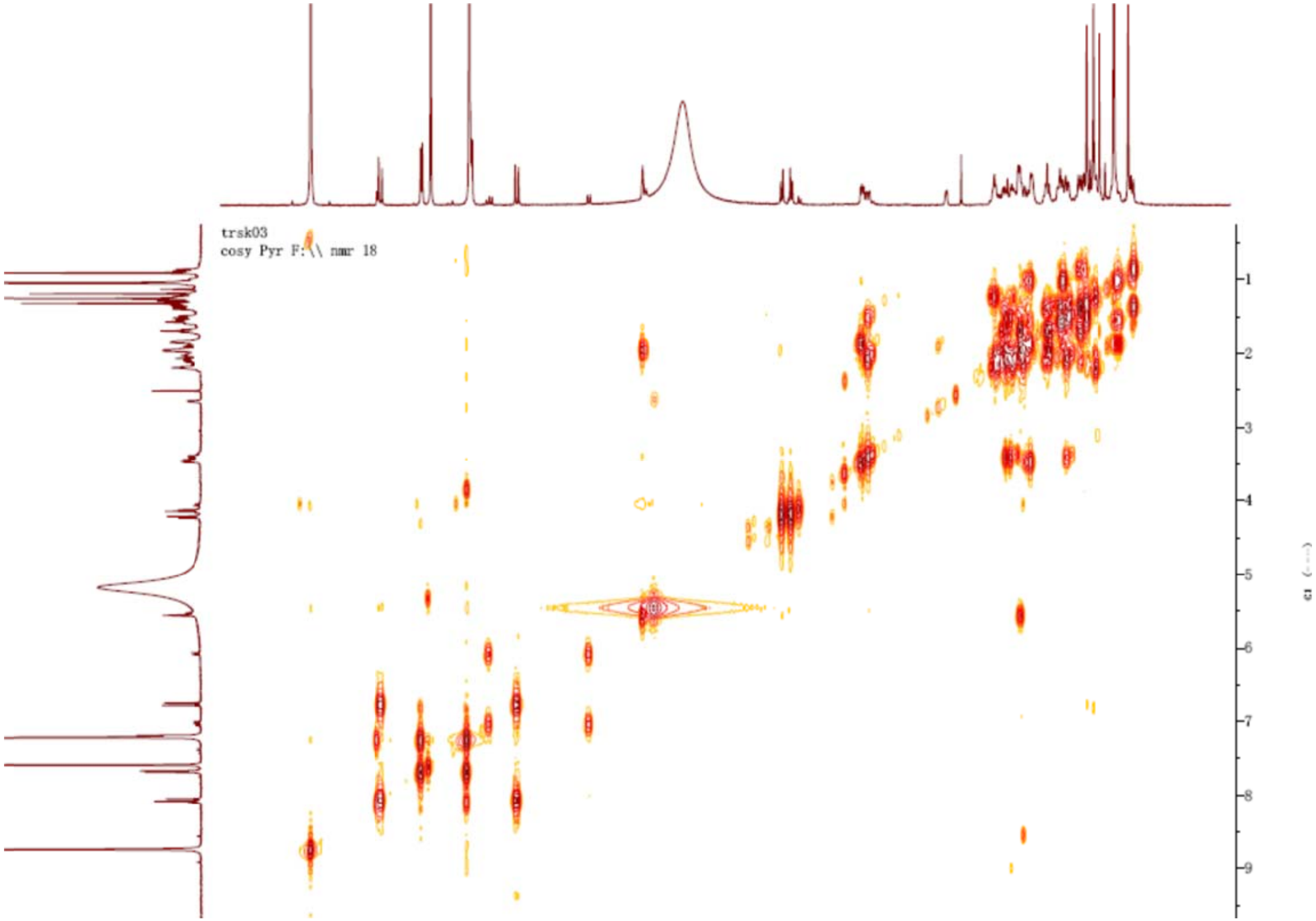

S16 The HMBC spectrum of 3*β*-hydroxy-olean-30-*p*-*E*-hydroxycinnamoyl-12-en-28-oic-acid (2)

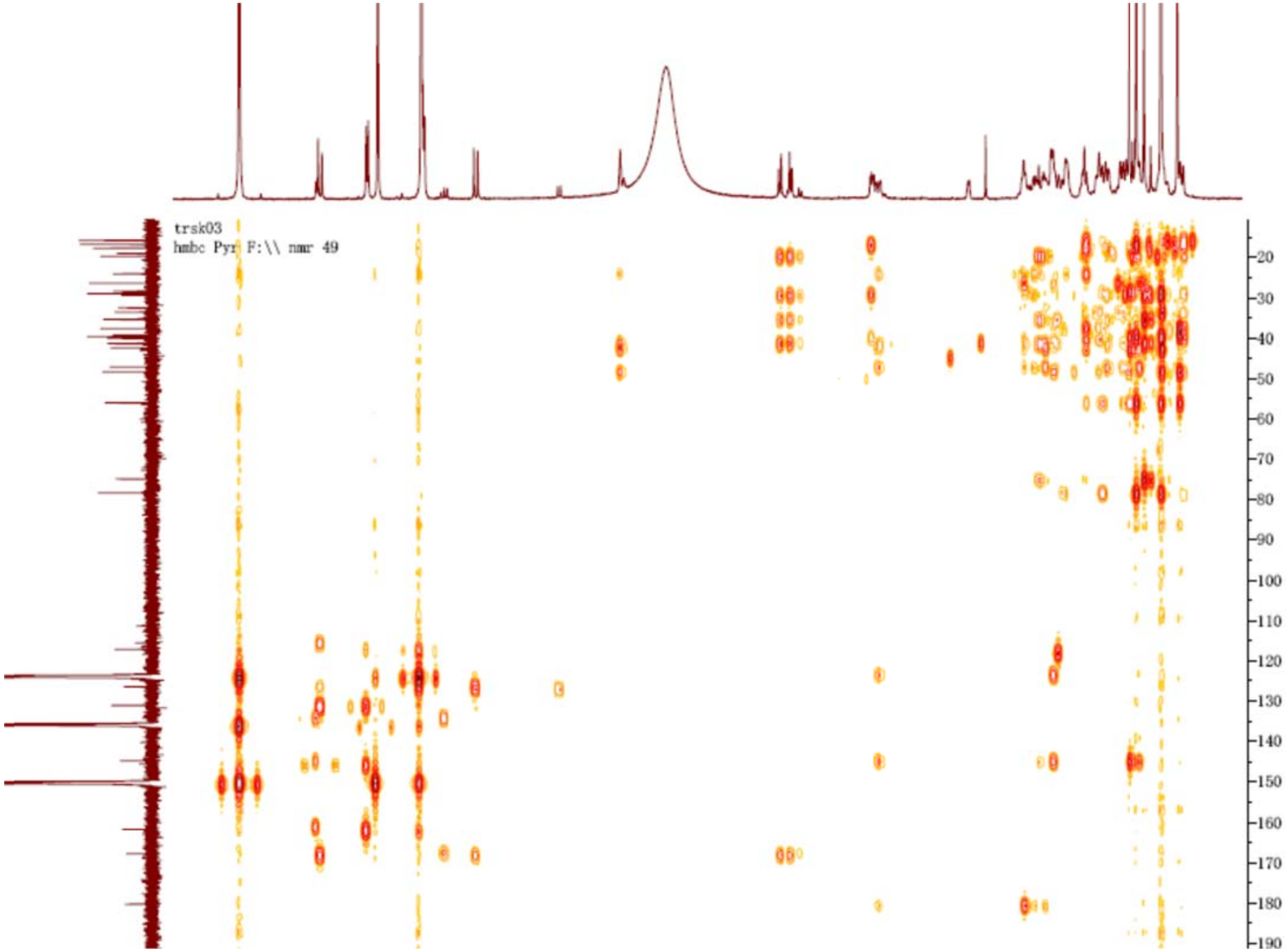

S17 The ROESY spectrum of 3*β*-hydroxy-olean-30-*p*-*E*-hydroxycinnamoyl-12-en-28-oic-acid (2)

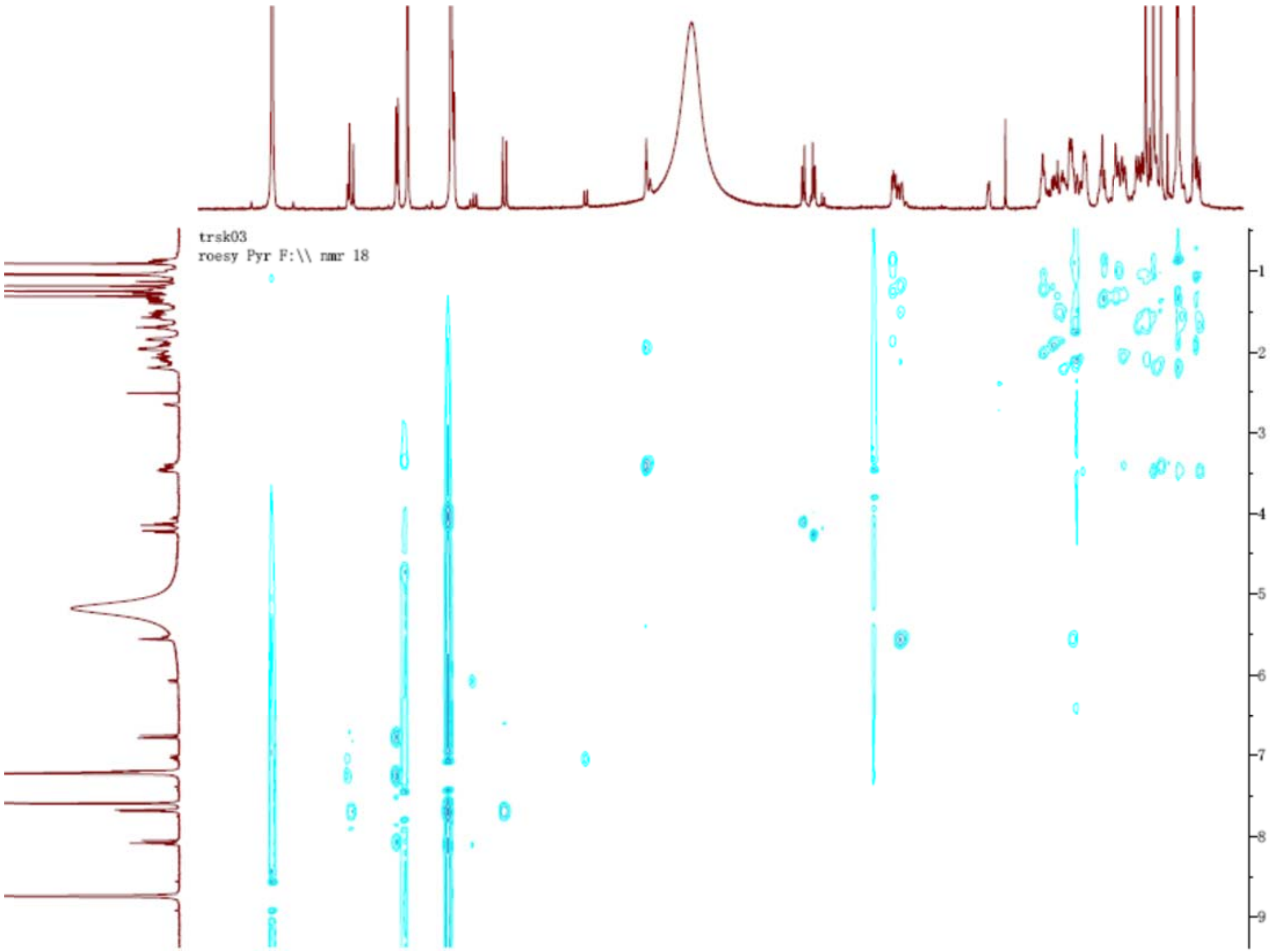

S18 The EIMS spectrum of 3*β*-hydroxy-olean-30-*p*-*E*-hydroxycinnamoyl-12-en-28-oic-acid (2)

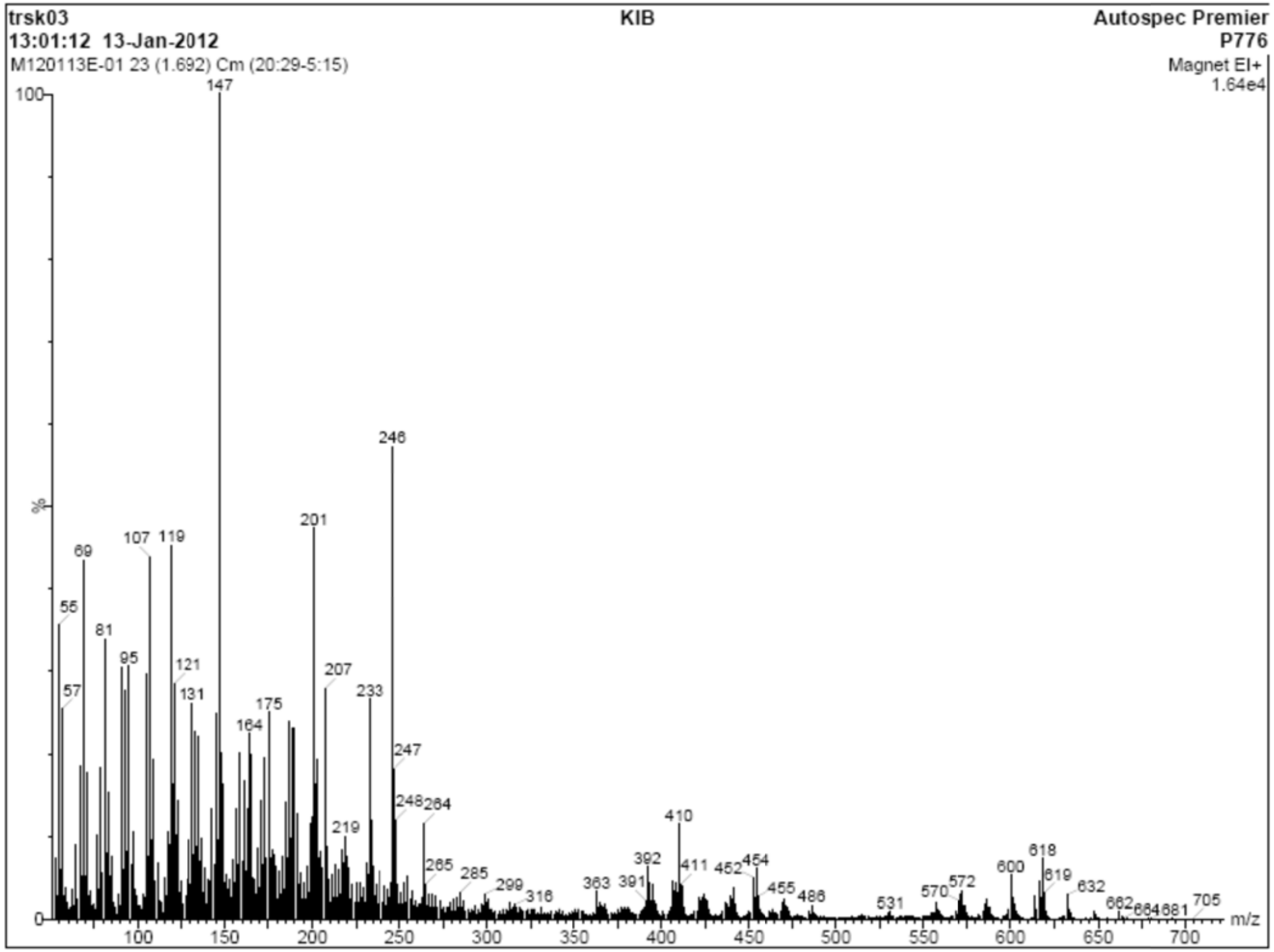

S19 The HREIMS spectrum of 3*β*-hydroxy-olean-30-*p*-*E*-hydroxycinnamoyl-12-en-28-oic-acid (2)

Single Mass Analysis (displaying only valid results)

Tolerance = 10.0 PPM / DBE: min = 0.5, max = 40.0

Selected filters: None

Monoisotopic Mass, Odd and Even Electron Ions
29 formula(e) evaluated with 1 results within limits (up to 51 closest results for each mass)
Elements Used:
C: 0-200 H: 0-400 O: 4-7

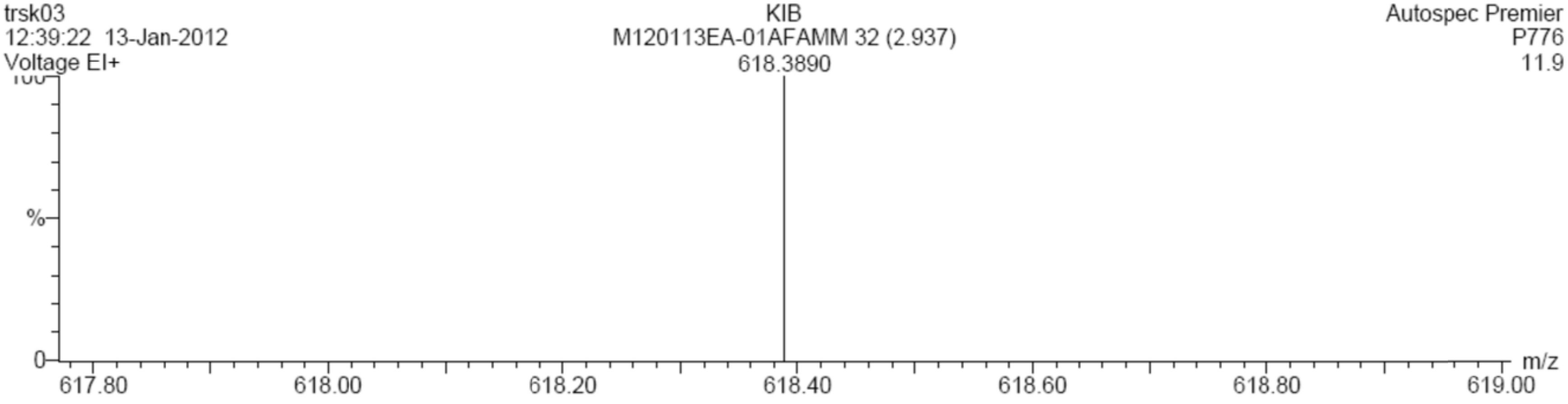

Minimum:				0.5		
Maximum:		100.0	10.0	40.0		
Mass	Calc. Mass	mDa	PPM	DBE	i-FIT	Formula
618.3890	618.3920	-3.0	-4.9	13.0	5546028.0	C39 H54 O6

S20 The IR spectrum of 3*β*-hydroxy-olean-30-*p*-*E*-hydroxycinnamoyl-12-en-28-oic-acid (2)

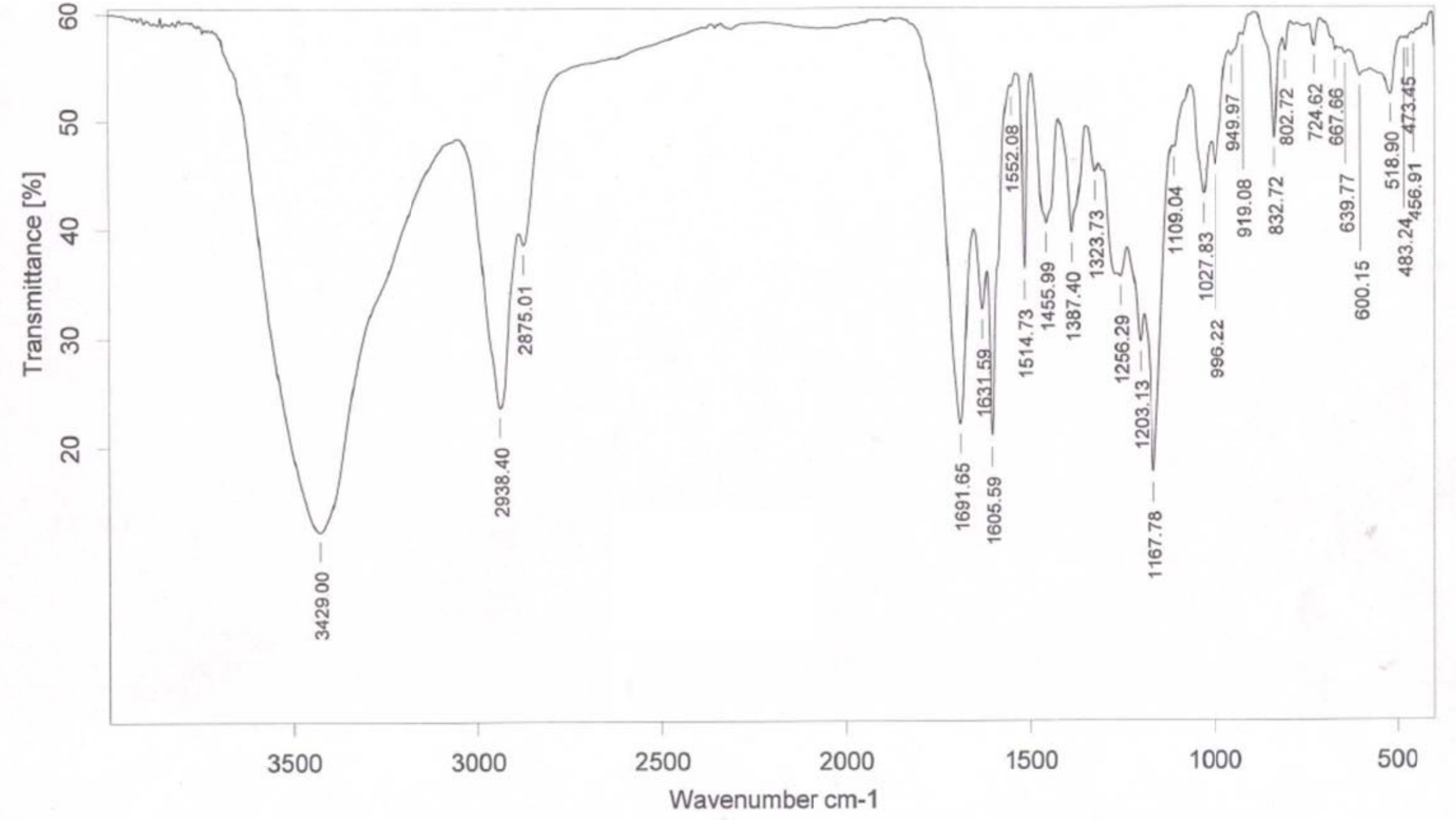

Sample : trsk03		Frequency Range : 399.246 - 3996.32	Measured on : 11/01/2012
Technique : KBr压片	Resolution : 4	Instrument : Tensor27	Sample Scans : 16
Customer : 120111IR7	Zerofilling : 2	Acquisition : Double Sided,For	

S21 The UV spectrum of 3*β*-hydroxy-olean-30-*p*-*E*-hydroxycinnamoyl-12-en-28-oic-acid (2)

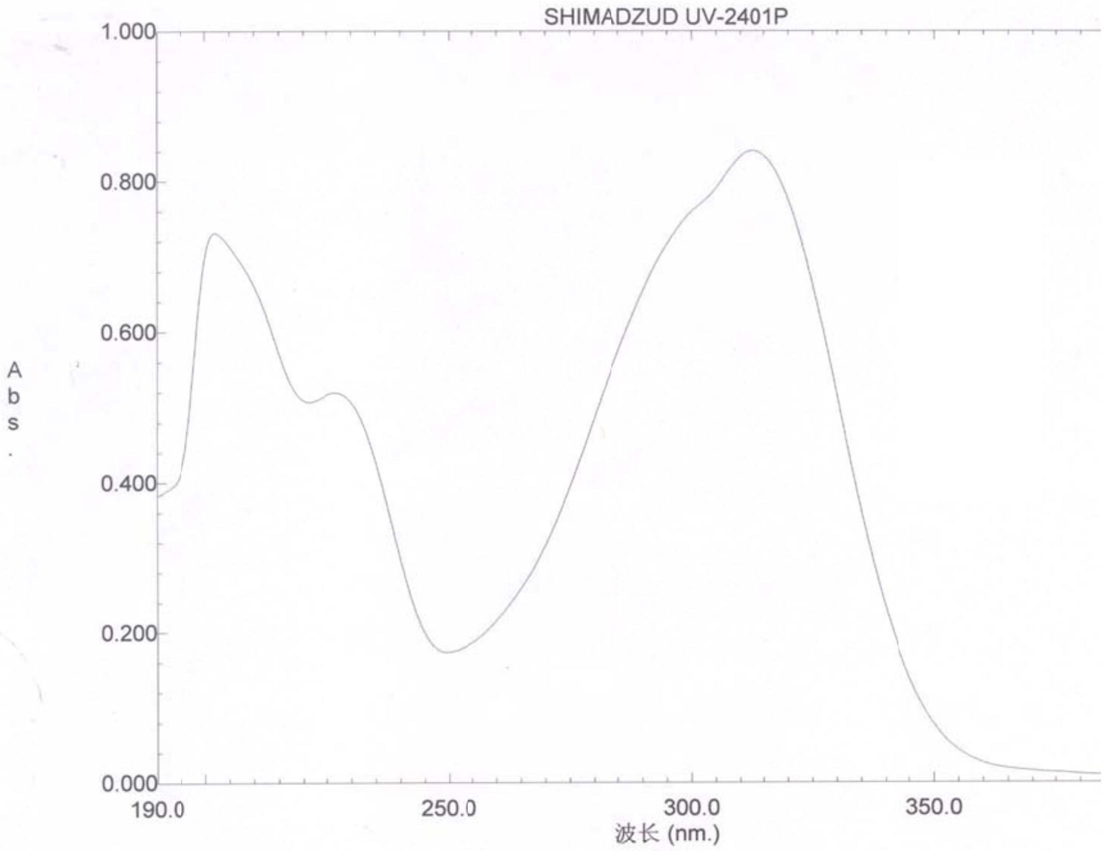

文件名: TRSK03

TRSK03 ———

样品浓度：0.0312毫克/毫升
溶剂：甲醇

创建于: 15:18 12-01-10
数据: 原始

测量模式: Abs.
扫描速度: 中速
狭缝: 5.0
采样间隔: 0.2

否.	波长 (nm.)	Abs.
1	391.40	0.0103
2	312.60	0.8403
3	226.80	0.5190
4	202.20	0.7312

S22 The $[\alpha]_D$ spectrum of 3*β*-hydroxy-olean-30-*p*-*E*-hydroxycinnamoyl-12-en-28-oic-acid (2)

Optical rotation measurement

Model : P-1020 (A060460638)

No.	Sample	Mode	Data	Monitor<br>Blank	Temp.<br>Cell<br>Temp Point	Date<br>Comment<br>Sample Name	Light<br>Filter<br>Operator	Cycle Time<br>Integ Time	
No.1	10 (1/3)	Sp.Rot	13.5430	0.0086<br>0.0000	16.3<br>50.00<br>Cell	Tue Jan 10 15:36:21 2012<br>0.00127g/mlMeOH<br>TRSK03	Na<br>589nm	2 sec<br>10 sec	
No.2	10 (2/3)	Sp.Rot	12.5980	0.0080<br>0.0000	16.3<br>50.00<br>Cell	Tue Jan 10 15:36:34 2012<br>0.00127g/mlMeOH<br>TRSK03	Na<br>589nm	2 sec<br>10 sec	+13.0709°
No.3	10 (3/3)	Sp.Rot	13.0710	0.0083<br>0.0000	16.3<br>50.00<br>Cell	Tue Jan 10 15:36:47 2012<br>0.00127g/mlMeOH<br>TRSK03	Na<br>589nm	2 sec<br>10 sec	

S23 The ^{1}H NMR spectrum of 3*β*, 6*α*-dihydroxy-urs-14-en-12-one (3) in C_5D_5N

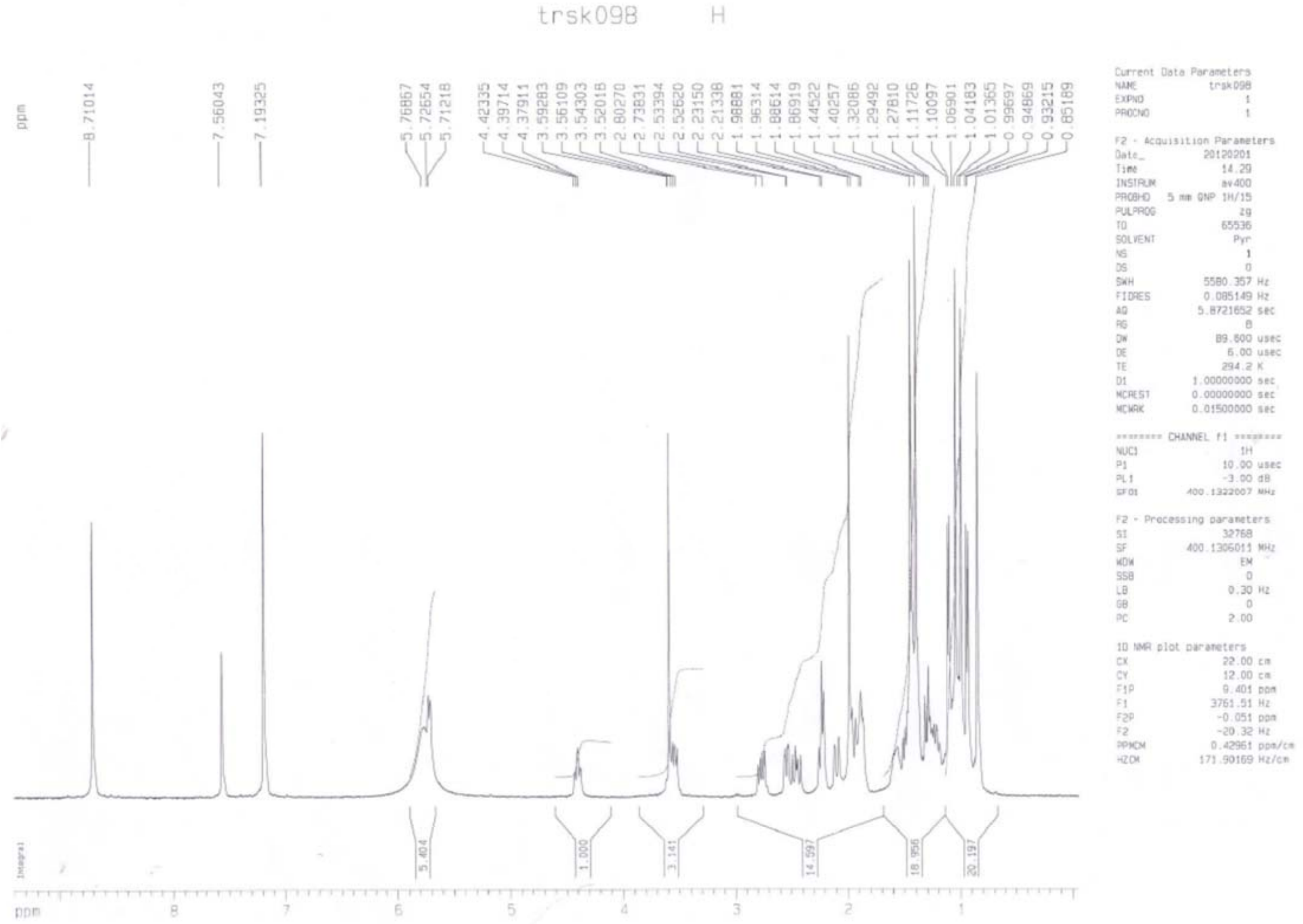

S24 The ^{13}C NMR spectrum of 3*β*, 6*α*-dihydroxy-urs-14-en-12-one (3) in C_5D_5N

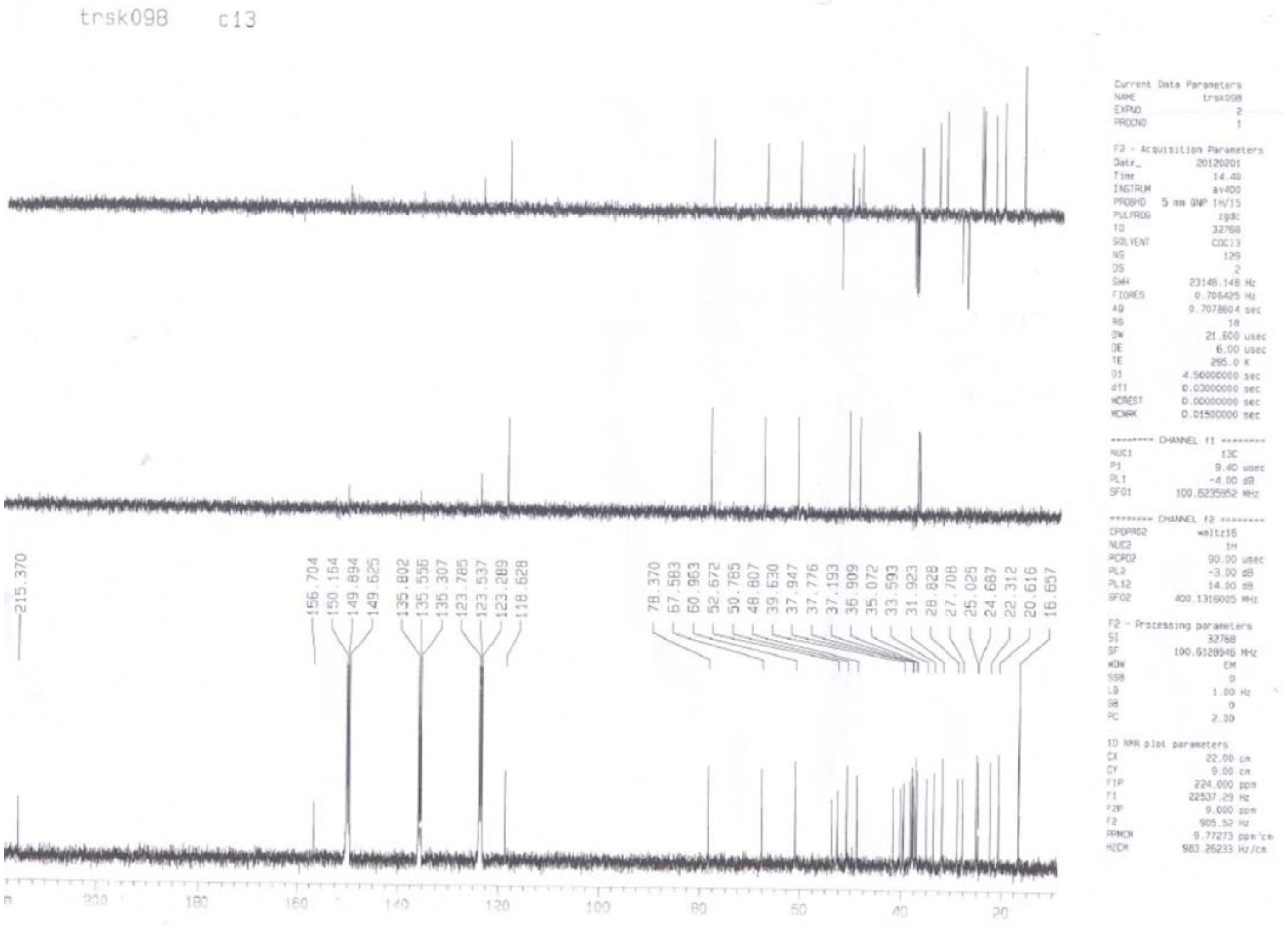

S25 The HSQC spectrum of 3*β*, 6*α*-dihydroxy-urs-14-en-12-one (3)

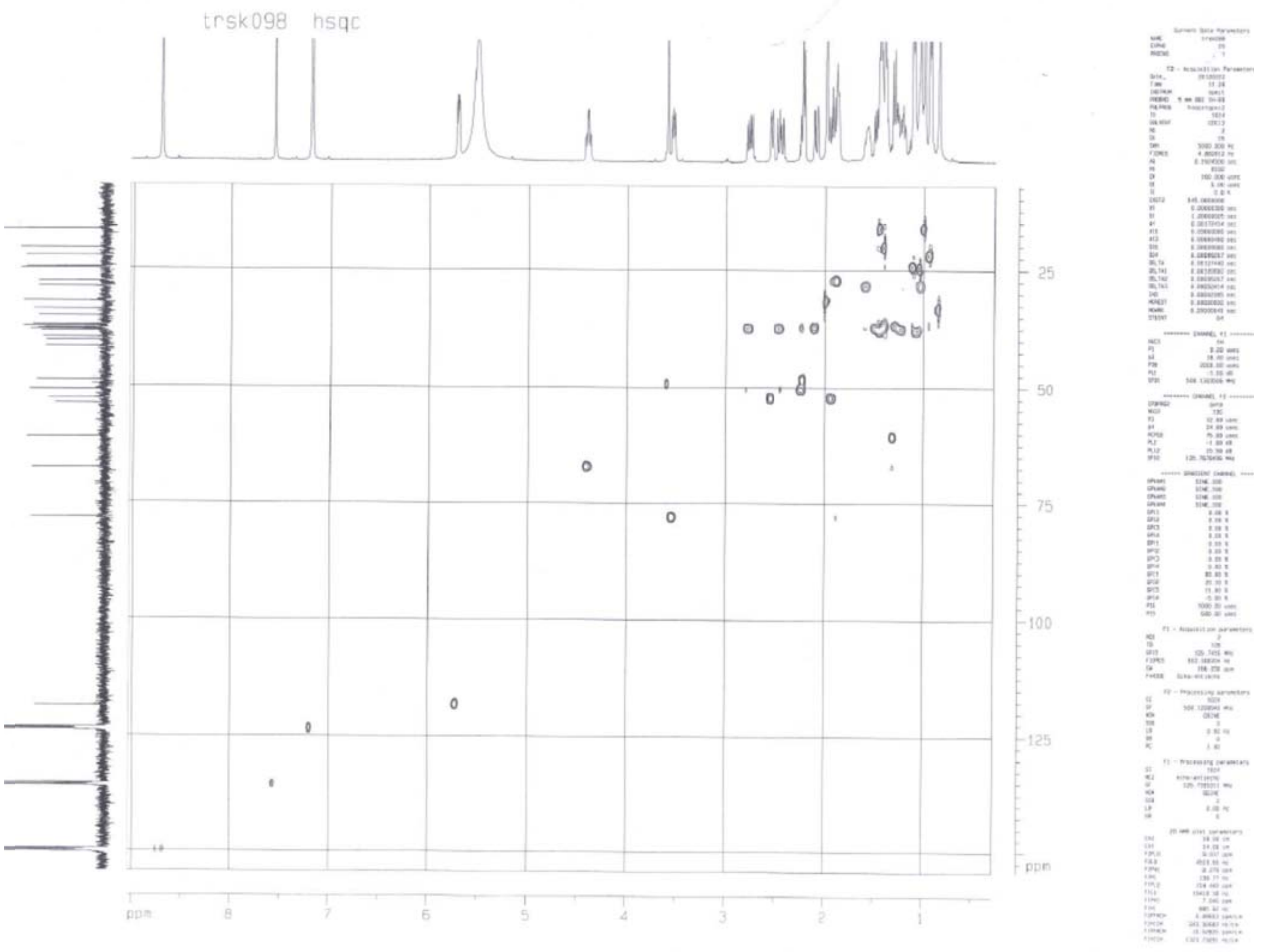

S26 The ^{1}H-^{1}H COSY spectrum of 3*β*, 6*α*-dihydroxy-urs-14-en-12-one (3)

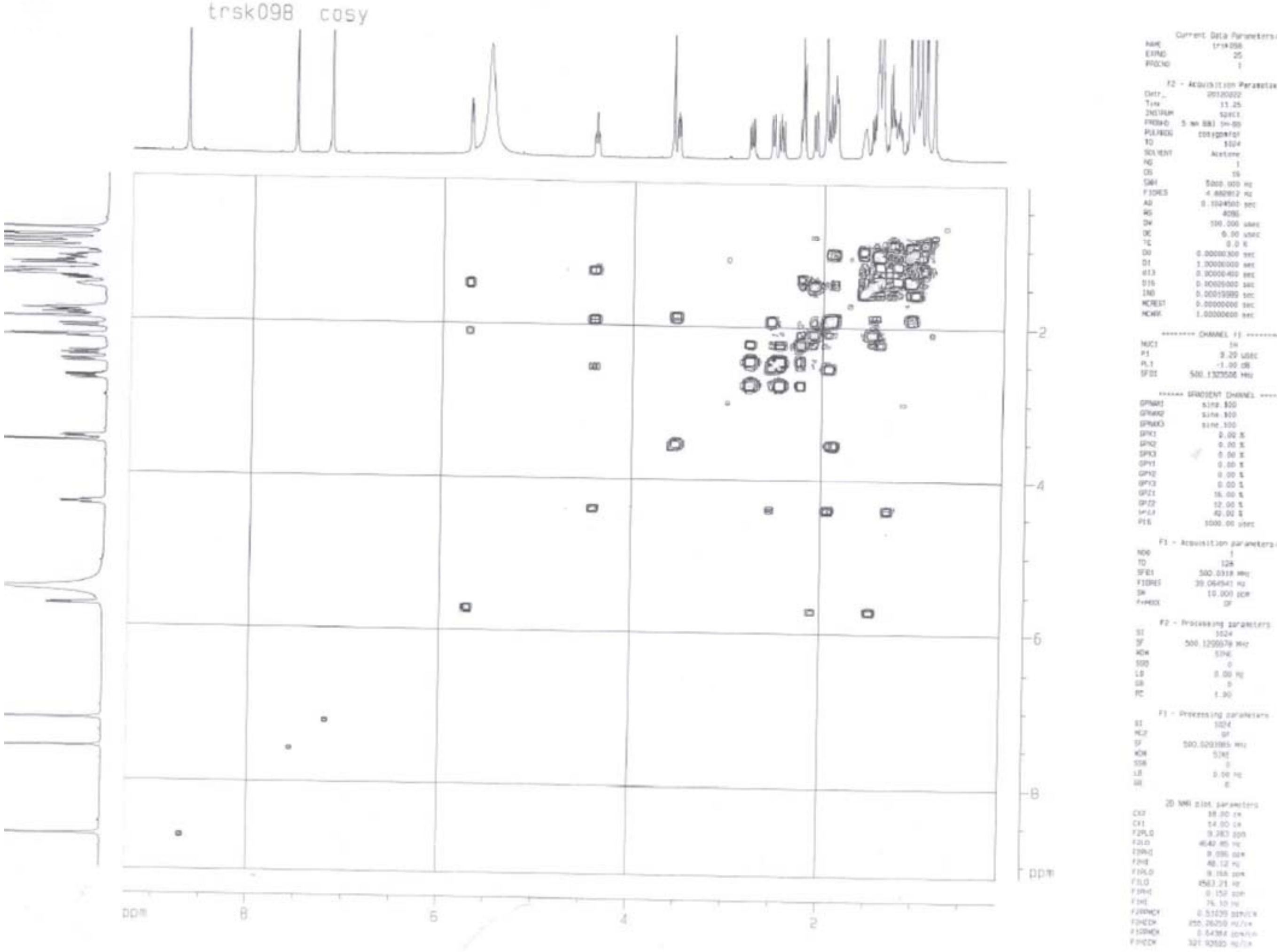

S27 The HMBC spectrum of 3*β*, 6*α*-dihydroxy-urs-14-en-12-one (3)

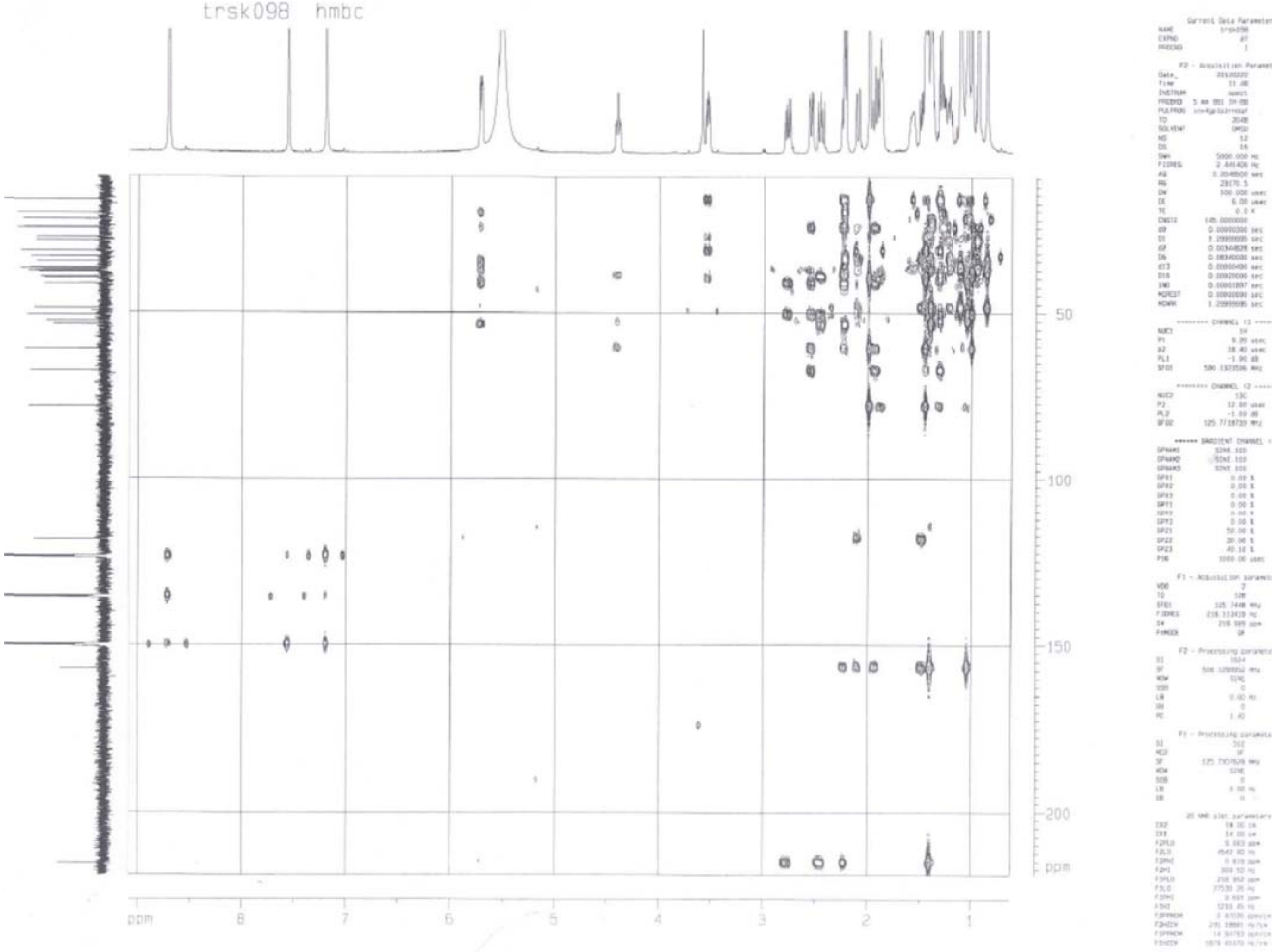

S28 The ROESY spectrum of 3*β*, 6*α*-dihydroxy-urs-14-en-12-one (3)

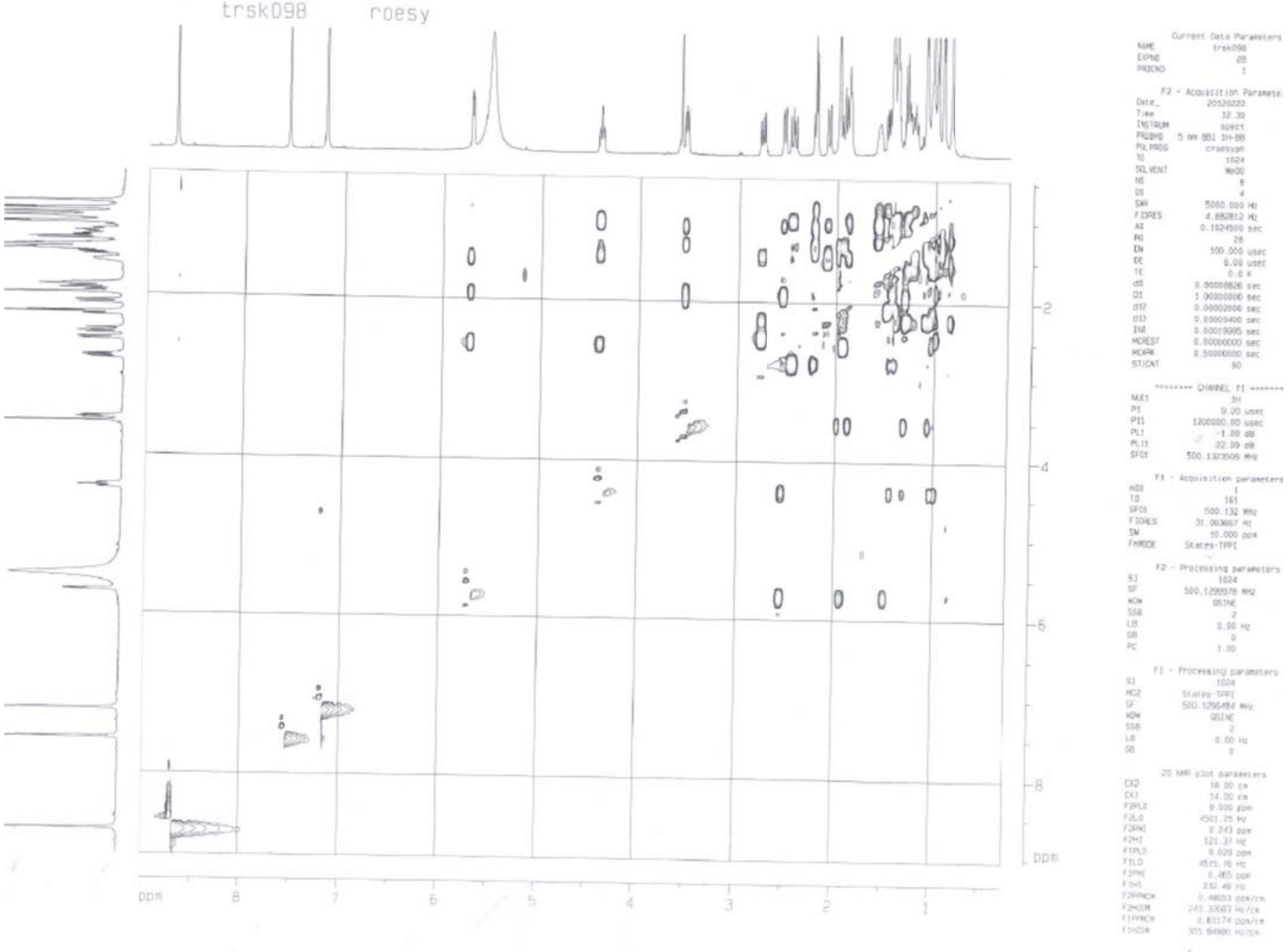

S29 The positive ESIMS spectrum of 3*β*, 6*α*-dihydroxy-urs-14-en-12-one (3)

Mass Spectrum List Report

Analysis Info

Analysis Name	D:\DATA\2012file\1202\120213\trsk09802.d	Acquisition Date	2/13/2012 3:43:45 PM
Method	ms_ptservice.m	Operator	Bruker
Sample Name	trsk098	Instrument	HCT

Acquisition Parameter

Ion Source Type	ESI	Ion Polarity	Positive	Alternating Ion Polarity	off
Mass Range Mode	Ultra Scan	Scan Begin	100 m/z	Scan End	1300 m/z
Capillary Exit	300.0 Volt	Skimmer	40.0 Volt	Trap Drive	70.0
Accumulation Time	617 µs	Averages	5 Spectra	Auto MS/MS	off

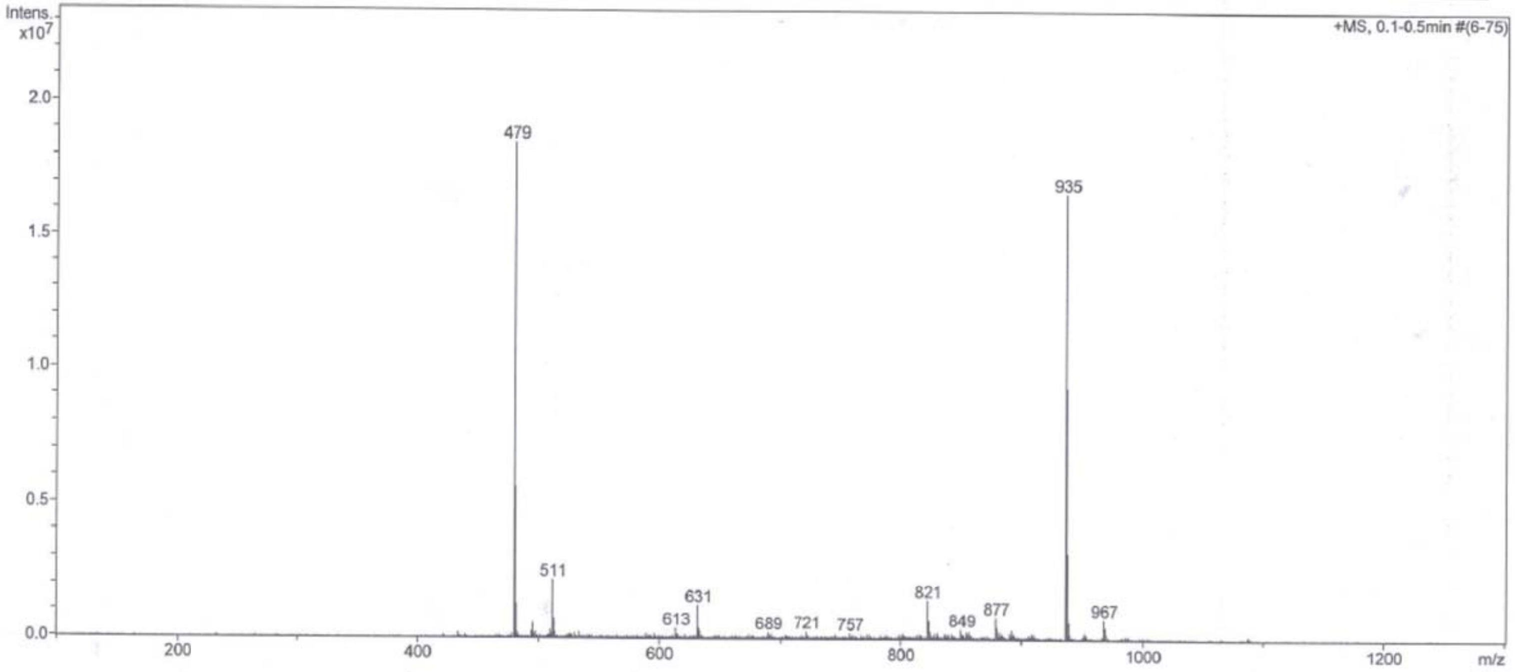

S30 a) The HRESIMS spectrum of 3*β*, 6*α*-dihydroxy-urs-14-en-12-one (3)

cq. Date: Friday, February 24, 2012 *Acq. Time: 15:31* *Scan Mode: Zero Width*

ample Name: 120224ESIA trsk098 *Sample Comment:*

Elemental composition calculator

Target m/z: +479.3504 amu
Tolerance: +10.0000 ppm
Result type: Elemental
Max num of results: 1000
Min DBE: -10.0000 Max DBE: +60.0000
Electron state: OddAndEven
Num of charges: 0
Add water:N/A
Add proton: N/A
File Name: 120224ESIA trsk098.wiff

	Elements	Min Number	Max Number
1	2H	0	0
2	Br	0	0
3	C	0	200
4	Cl	0	0
5	F	0	0
6	H	0	400
7	I	0	0
8	K	0	0
9	N	0	0
10	Na	1	1
11	O	1	4

S30 b) The HRESIMS spectrum of 3*β*, 6*α*-dihydroxy-urs-14-en-12-one (3)

cq. Date: Friday, February 24, 2012 Acq. Time: 15:31 Scan Mode: Zero Width

ample Name: 120224ESIA trsk098 Sample Comment:

	Elements	Min Number	Max Number
12	P	0	0
13	Pt	0	0
14	S	0	0
15	Si	0	0

	Formula	Calculated m/z (amu)	mDa Error	PPM Error	DBE
1	C30 H48 O3 Na	479.3501	0.2844	0.5934	6.5

S31 The IR spectrum of 3*β*, 6*α*-dihydroxy-urs-14-en-12-one (3)

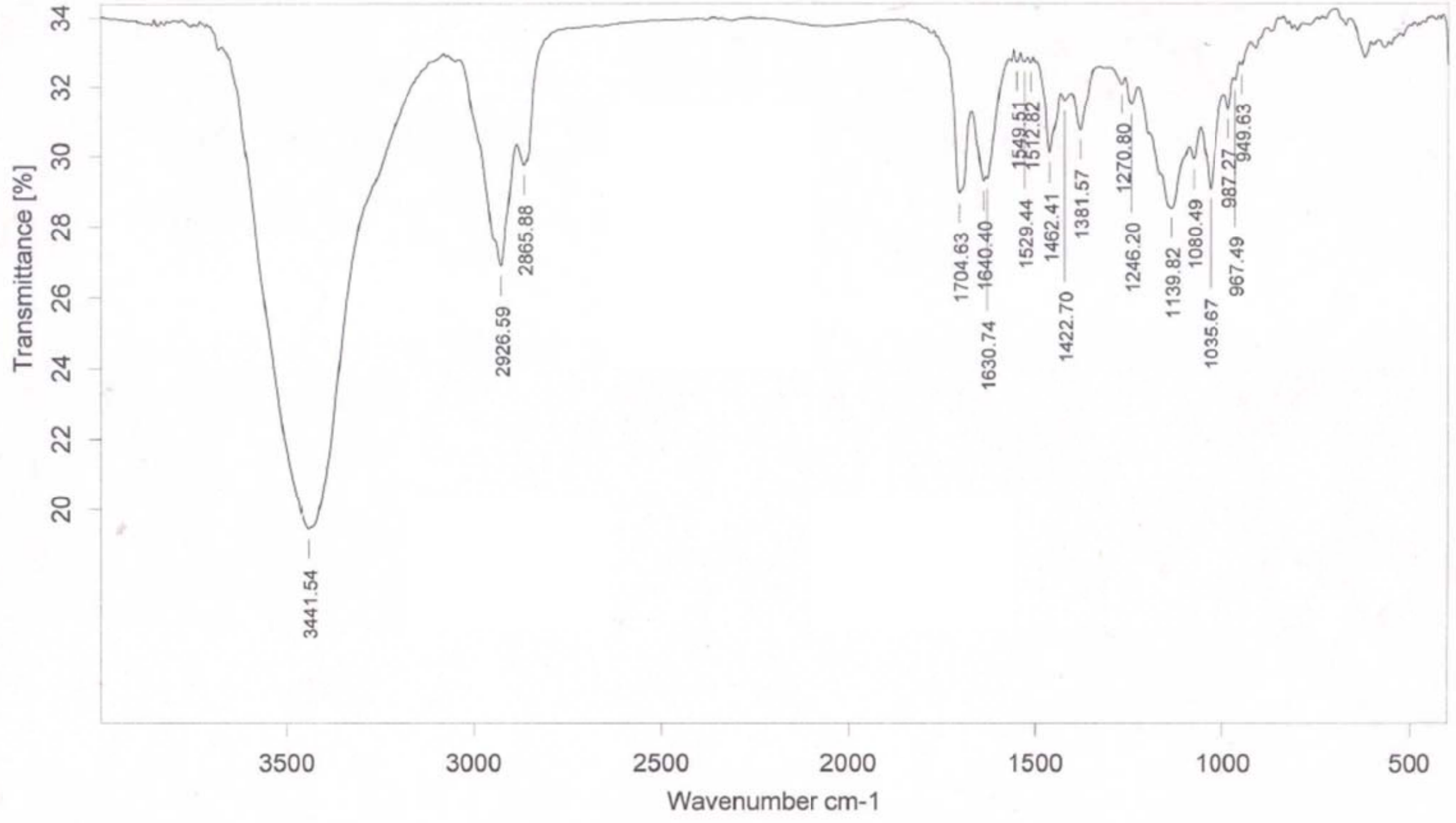

Sample : trsk098	Frequency Range : 399.246 - 3996.32		Measured on : 24/02/2012
Technique : KBr压片	Resolution : 4	Instrument : Tensor27	Sample Scans : 16
Customer : 120224IR2	Zerofilling : 2	Acquisition : Double Sided,For	

S32 The UV spectrum of 3β, 6α-dihydroxy-urs-14-en-12-one (3)

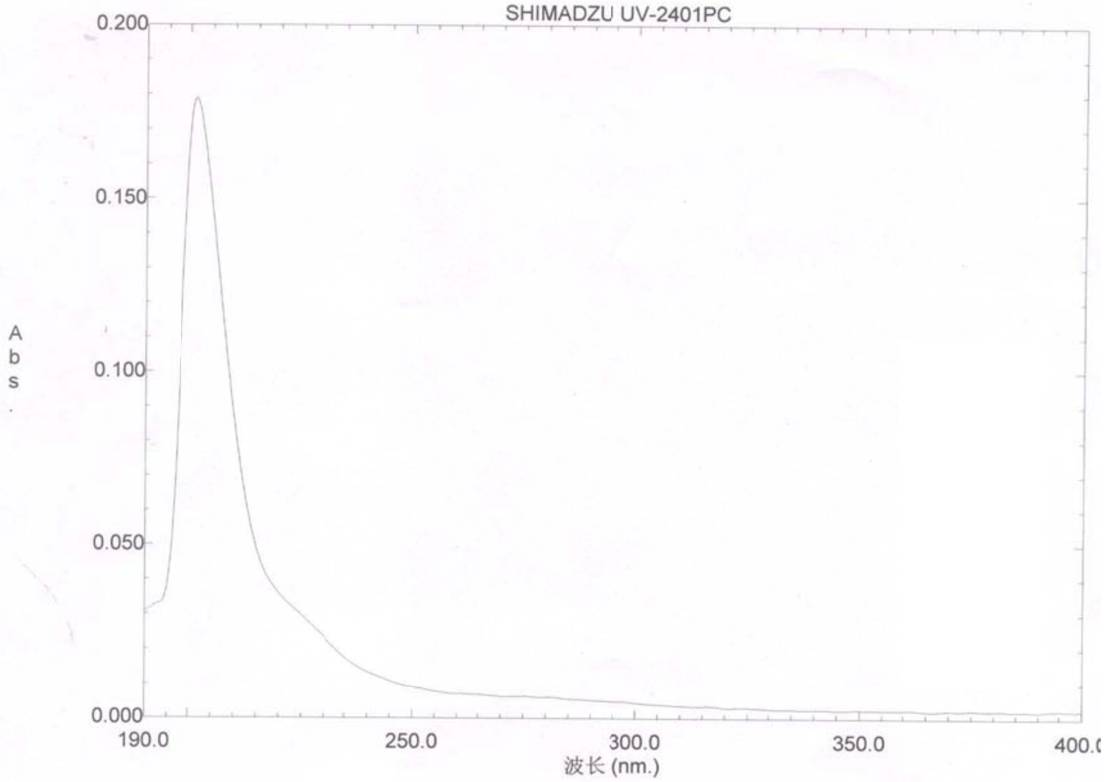

文件名: TRSK098

TRSK098

创建于: 15:53 12-02-24
数据: 原始

样品浓度：0.0320毫克/毫升
溶剂：甲醇

测量模式: Abs.
扫描速度: 中速
狭缝: 5.0
采样间隔: 0.2

否.	波长 (nm.)	Abs.
1	201.40	0.1789

S33 The $[\alpha]_D$ spectrum of 3*β*, 6*α*-dihydroxy-urs-14-en-12-one (3)

Optical rotation measurement

Model : P-1020 (A060460638)

No.	Sample	Mode	Data	Monitor<br>Blank	Temp.<br>Cell<br>Temp Point	Date<br>Comment<br>Sample Name	Light<br>Filter<br>Operator	Cycle Time<br>Integ Time
No.1	6 (1/3)	Sp.Rot	-11.8000	-0.0059<br>0.0000	20.1<br>50.00<br>Cell	Fri Feb 24 15:57:06 2012<br>0.00100g/mlMeOH<br>TRSK098	Na<br>589nm	2 sec<br>10 sec
No.2	6 (2/3)	Sp.Rot	-10.6000	-0.0053<br>0.0000	20.0<br>50.00<br>Cell	Fri Feb 24 15:57:19 2012<br>0.00100g/mlMeOH<br>TRSK098	Na<br>589nm	2 sec<br>10 sec
No.3	6 (3/3)	Sp.Rot	-9.8000	-0.0049<br>0.0000	20.0<br>50.00<br>Cell	Fri Feb 24 15:57:32 2012<br>0.00100g/mlMeOH<br>TRSK098	Na<br>589nm	2 sec<br>10 sec

−10.7333°
